# Activation of the alpha-globin gene expression correlates with dramatic upregulation of nearby non-globin genes and changes in local and large-scale chromatin spatial structure

**DOI:** 10.1186/s13072-017-0142-4

**Published:** 2017-07-11

**Authors:** Sergey V. Ulianov, Aleksandra A. Galitsyna, Ilya M. Flyamer, Arkadiy K. Golov, Ekaterina E. Khrameeva, Maxim V. Imakaev, Nezar A. Abdennur, Mikhail S. Gelfand, Alexey A. Gavrilov, Sergey V. Razin

**Affiliations:** 1 Institute of Gene Biology of the Russian Academy of Sciences, Moscow, Russia 119334; 2 Faculty of Biology, M.V. Lomonosov Moscow State University, Moscow, Russia 119992; 3 Faculty of Bioengineering and Bioinformatics, M.V. Lomonosov Moscow State University, Moscow, Russia 119992; 4 Skolkovo Institute of Science and Technology, Skolkovo, Russia 143026; 5 Institute for Information Transmission Problems (the Kharkevich Institute) of the Russian Academy of Sciences, Moscow, Russia 127051; 6 Department of Physics, Massachusetts Institute of Technology, Cambridge, MA 02139 USA; 7 Computational and Systems Biology Graduate Program, Massachusetts Institute of Technology, Cambridge, MA USA; 8 Faculty of Computer Science, Higher School of Economics, Moscow, Russia 125319; 9 MRC Human Genetics Unit, Institute of Genetics and Molecular Medicine, University of Edinburgh, Edinburgh, UK

**Keywords:** Alpha-globin genes, Transcription, CTCF, Chromatin spatial structure, TAD, Chromatin compartment

## Abstract

**Background:**

In homeotherms, the alpha-globin gene clusters are located within permanently open genome regions enriched in housekeeping genes. Terminal erythroid differentiation results in dramatic upregulation of alpha-globin genes making their expression comparable to the rRNA transcriptional output. Little is known about the influence of the erythroid-specific alpha-globin gene transcription outburst on adjacent, widely expressed genes and large-scale chromatin organization. Here, we have analyzed the total transcription output, the overall chromatin contact profile, and CTCF binding within the 2.7 Mb segment of chicken chromosome 14 harboring the alpha-globin gene cluster in cultured lymphoid cells and cultured erythroid cells before and after induction of terminal erythroid differentiation.

**Results:**

We found that, similarly to mammalian genome, the chicken genomes is organized in TADs and compartments. Full activation of the alpha-globin gene transcription in differentiated erythroid cells is correlated with upregulation of several adjacent housekeeping genes and the emergence of abundant intergenic transcription. An extended chromosome region encompassing the alpha-globin cluster becomes significantly decompacted in differentiated erythroid cells, and depleted in CTCF binding and CTCF-anchored chromatin loops, while the sub-TAD harboring alpha-globin gene cluster and the upstream major regulatory element (MRE) becomes highly enriched with chromatin interactions as compared to lymphoid and proliferating erythroid cells. The alpha-globin gene domain and the neighboring loci reside within the A-like chromatin compartment in both lymphoid and erythroid cells and become further segregated from the upstream gene desert upon terminal erythroid differentiation.

**Conclusions:**

Our findings demonstrate that the effects of tissue-specific transcription activation are not restricted to the host genomic locus but affect the overall chromatin structure and transcriptional output of the encompassing topologically associating domain.

**Electronic supplementary material:**

The online version of this article (doi:10.1186/s13072-017-0142-4) contains supplementary material, which is available to authorized users.

## Background

In mammals, tissue-specific genes are often located within permanently active chromosome regions, which are enriched with genes ubiquitously transcribed in various cell types. The current paradigm suggests that tissue-specific transcriptional regulation within complex genome environment depends on a specific spatial organization of the interphase chromatin [1–3]. Non-random long-range contacts between remote regulatory sequences and promoters in mammalian genomes are often anchored by CTCF/cohesin-mediated chromatin loops [4] and preferentially occur within the same topologically associating domain (TAD) [5, 6]. TAD boundaries were found to be important for preventing abnormal enhancer–promoter communication [7, 8] and, consequently, delimiting zones of “licensed” enhancer influence, or regulatory domains [9].

The alpha-globin gene domain (AgGD) is defined here as a cluster of α-globin genes along with the remote regulatory elements. The AgGDs of warm-blooded vertebrates represent a canonical and arguably the most comprehensively studied example of a genomic locus where an array of tissue-specific (erythroid) genes is located within an extended cluster of housekeeping genes [10, 11]. The structure of AgGD is highly conserved among homeotherms. The domain comprises several alpha-globin genes transcribed in erythroid cells in a developmental stage-specific manner, and several enhancers whose exact number may vary slightly in different taxa [11]. A relatively long genomic region upstream of AgGD is syntenic in vertebrates [12]. The major regulatory element (MRE) of the AgGD, a strong and evolutionary conserved erythroid-specific enhancer [13, 14], is obligatorily located within an intron of the gene *NPRL3* residing immediately upstream of the alpha-globin cluster. Although the AgGD is located in a permanently open (DNAse I-sensitive) highly acetylated chromatin in all cell types [15], full activation of the alpha-globin transcription in differentiated erythroid cells is accompanied by further histone hyperacetylation [16, 17] and reconfiguration of the local chromatin spatial structure within the domain. In these cells, MRE interacts with the adult alpha-globin gene promoters via direct looping, and this interaction is a prerequisite for proper development stage-specific expression of the adult alpha-globin genes [18, 19].

Previous studies have revealed that full activation of the AgGD in erythroid cells of different origin also has certain distant effects on surrounding transcription and chromatin structure. In primary human erythroid cells, the active status of the alpha-globin genes correlates with upregulation of the nearby *NPRL3* gene and the distant non-related gene *NME4*, located 220 Kb away, which is activated via spatial interaction with the MRE [20]. In the human malignant erythroid cell line K562, as compared to lymphoid cells, transcription of the alpha-globin genes correlates with the decompaction of a 500-Kb chromosomal segment harboring the alpha-globin gene cluster, based on 5C data and polymer simulations [21]. In mouse primary erythroid cells, active alpha-globin genes may be recruited to a transcription factory formed by promoters of housekeeping genes located up to 70 Kb away [22]. Thus, it appears that the activation of globin genes and the assembly of the alpha-globin active chromatin hub occur simultaneously with the modification of the spatial structure of a genomic region at least several hundred kilobases in size.

Here, to get a systematic view on the effects of high-output AgGD activation on neighboring gene loci and chromatin structure, we studied a 2.7 Mb-region of chicken chromosome 14 harboring AgGD, 35 non-globin (predominantly housekeeping) genes, and a gene desert. We performed total rRNA-depleted transcriptome profiling to capture both genic and intergenic transcription, and applied high-resolution 3C, a new variant of Capture Hi-C (Chromatin TArget Ligation Enrichment, C-TALE), and large-scale 5C approaches coupled with a high-throughput analysis of CTCF binding to probe the spatial organization of the genome at the levels of CTCF-anchored loops, TADs, and compartments. We have found that transcription outburst of the alpha-globin genes in terminally differentiated erythroblasts is accompanied by (i) a substantial increase in the level of transcription of several adjacent housekeeping genes and intergenic regions, (ii) dramatic compaction of the encompassing sub-TAD amid the chromatin decompaction and massive attenuation of CTCF binding within the extended chromosome vicinity, and (iii) large-scale changes in the chromatin interaction profile at the level of chromatin compartments including segregation of AgGD from the transcriptionally silent gene desert. Our results suggest that activation of tissue-specific transcription may considerably affect adjacent non-related genes and chromatin folding of a large chromosomal segment. The opposite scenario where reconfiguration of an extended genomic domain enables remodeling of the local chromatin structure within AgGD also cannot be ruled out.

## Results

### Full activation of the alpha-globin gene expression is accompanied by the emergence of abundant intergenic transcription and upregulation of nearby non-globin genes

We used three cell types—cultured lymphoid DT40 cells, which are not expressing globins, and cultured erythroid HD3 cells that were either proliferating (committed to globin expression) or differentiated (actively expressing globins). To precisely track changes in the transcriptional profile during terminal erythroid differentiation, we performed sequencing of total rRNA-depleted transcriptomes of these cell types. Of note, although chicken erythroblasts are extensively used for studies of the genome biology and function [23–26], a whole-transcriptome analysis and comparison with lymphoblasts and between the differentiation stages, to our knowledge, has not been reported previously.

**Fig. 1:**
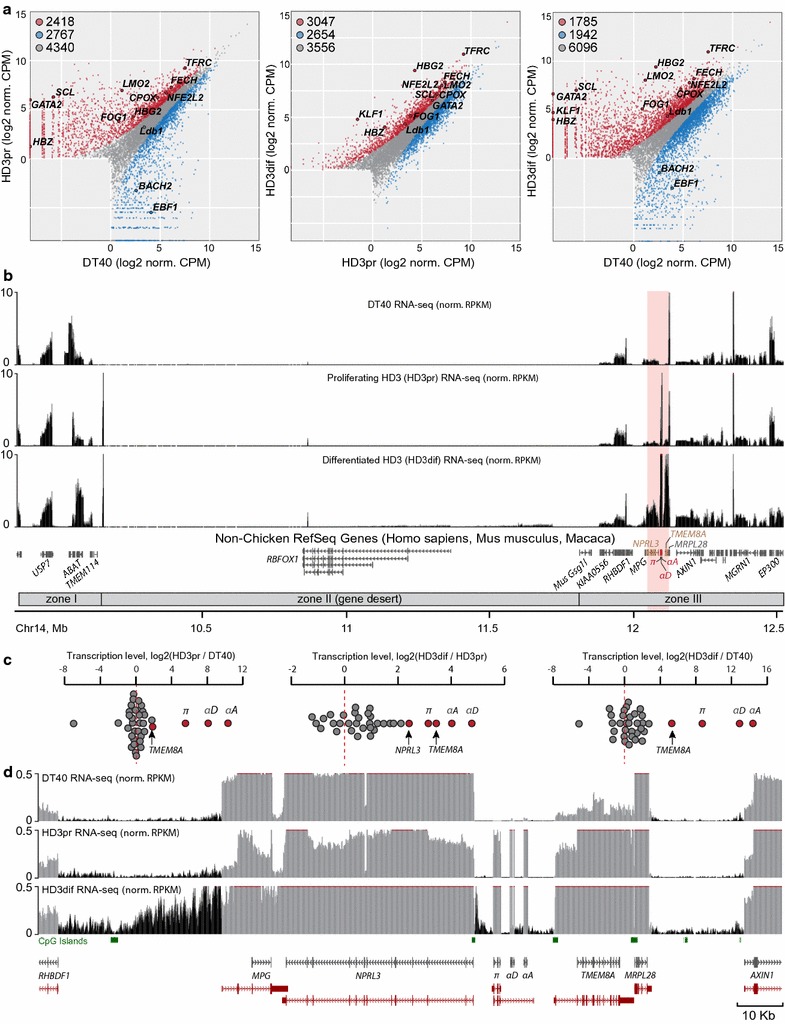
Analysis of transcriptomes of the studied cell types. **a** Genome-wide variation of gene expression in the studied cell types (proliferating and differentiated HD3 cells are designated by HD3pr and HD3dif, respectively). Numbers of upregulated, downregulated, and ubiquitously transcribed genes for each pair of the cell types are shown at the *upper left corner* of the plots. The following genes are highlighted in the plots: (1) erythroid transcription factors *GATA2*, *SCL* (*Tal1*), *FOG1*, *LMO2*, *NF*-*E2*, *Ldb1* and *KLF1* (*EKLF*); (2) enzymes involved in heme synthesis including *FECH* and *CPOX*; (3) transferrin receptor (*TFRC)*; and (4) the alpha-globin gene *π* (*HBZ)* and beta-globin gene (*HBG2)*. *BACH2* and *EBF1* are lymphoid transcription factors. **b** Normalized profiles of total rRNA-depleted RNA-seq within the studied region. The functional AgGD from the 3′-end of the *NPRL3* gene to the 3′-end of the *TMEM8A* gene is highlighted in *pink*. Alpha-globin genes are highlighted in *red*, and non-globin genes located within the AgGD are highlighted in *brown*. **c** Transcription level changes between cell types for all genes within the studied region. **d** The RNA-seq profile of the AgGD and closest neighbors. Intergenic transcription profiles are highlighted in *black*, and genic transcription profiles are highlighted in *gray*. Positions of genes from the Ensembl database are highlighted in *red*

We performed cluster analysis, differential expression analysis and gene ontology annotation, and expectedly found that genome-wide differences in the transcriptional profile are more pronounced between HD3 and DT40 cells than between proliferating and differentiated HD3 cells (Fig. 1a; Additional file 1: Figure S1, panels a, b and c). The differential expression analysis reveals the upregulation (logFC > −0.6, FDR < 10^−7^) of numerous erythroid genes in differentiated HD3 cells (Fig. 1a), including genes encoding transcription factors (*KLF1*, *GATA2*, *SCL*, *LMO2*, *NF*-*E2* and *FOG1*), enzymes involved in heme synthesis (mitochondrial ferrochelatase [*FECH*] and coproporphyrinogen oxidase [*CPOX*]) and cell surface markers (transferrin receptor [*TFRC*]). Gene ontology annotation reveals that the differentiation of HD3 cells is accompanied by a significant decrease in the expression of metabolism-related genes (*P* < 10^−18^, Additional file 1: Figure S1d), indicating total repression of cellular biosynthetic processes, which recapitulates the normal erythrocyte maturation [27].

The studied genomic region harbors two gene-rich, highly transcribed zones (5′-terminal zone I and 3′-terminal zone III containing AgGD) separated by a 1.6-Mb gene desert (zone II) containing a predicted homologue of the human gene *RBFOX1* (Fig. 1b). In differentiated HD3 cells, about 50% of genes in the entire studied region display higher expression as compared to DT40 and proliferating HD3 cells (logFC > 0.6, FDR < 10^−7^; Fig. 1; see normalized RNA-seq profiles in panel [b]). Importantly, non-globin genes, *NPRL3* and *TMEM8A*, located within the AgGD and involved in the formation of the alpha-globin active chromatin microcompartment (or active chromatin hub, ACH) [26, 28], show the highest degree of upregulation among non-globin genes in differentiated HD3 cells (5.4-fold and 10.8-fold, respectively; Fig. 1c; Additional file 2: Table S1).

A remarkable feature of the transcriptome profile in the vicinity of AgGD is the presence of abundant intergenic transcription, especially in differentiated HD3 cells (Fig. 1d). The highest level of intergenic transcription is observed for regions between the *RHBDF1* promoter and the *MPG* promoter, and between *MRPL28* and *AXIN1* genes, i.e., within immediate neighborhoods of the AgGD. Within the alpha-globin gene cluster, intergenic transcription is detected exclusively in differentiated HD3 cells. In these cells, previous studies using RT-PCR and Northern blotting have demonstrated the presence of the so-called alpha-globin full-domain transcript, a long noncoding RNA presumably covering the region from its promoter within the *NPRL3* gene to the 3′-enhancer of AgGD [29–31]. Indeed, our RNA-seq data reveal the presence of low-level intergenic transcription from the *NPRL3* promoter to the *HBZ* (*π*) gene, and between *HBAD* (*α* ^*D*^) and *HBAA* (*α* ^*A*^) genes (Fig. 1d) in differentiated HD3 cells. Furthermore, in these cells, intergenic transcription is readily detectable between the 3′-enhancer of AgGD and the *TMEM8A* promoter. Systematic analysis reveals that transcription level within intergenic regions is substantially increased in differentiated HD3 cells genome-wide (Additional file 1: Figure S1e), the six intergenic regions from an immediate vicinity of AgGD being among the top 5% most upregulated.

Taken together, our data reveal general transcriptional upregulation and the presence of abundant intergenic transcription within and around AgGD in differentiated HD3 cells.

### AgGD is located within A-like chromatin compartment in both lymphoid and erythroid cells, and is further spatially segregated from the gene desert upon erythroid differentiation

Transcription regulation and changes in chromatin epigenetic state upon erythroid differentiation were extensively studied in warm-blooded vertebrate alpha-globin gene domains [10, 11, 32–35]. However, large-scale spatial chromatin structure was precisely examined only in human AgGD [21]. Here, we performed 5C analysis of an extended region of the chicken chromosome 14 that harbors the AgGD. The experiments were conducted in two biological replicates for each cell type as described previously [36] with minor modifications (see “Methods” and Additional file 3: Figure S2). A total of 93 forward primers and 92 reverse primers were used (on average, one primer per 14.5 Kb, Additional file 4: Table S2), and 8556 unique pairwise chromatin interactions were detected within the selected area. The raw 5C data were binned at a 30 Kb resolution, iteratively corrected, and smoothed (see “Methods”).

**Fig. 2:**
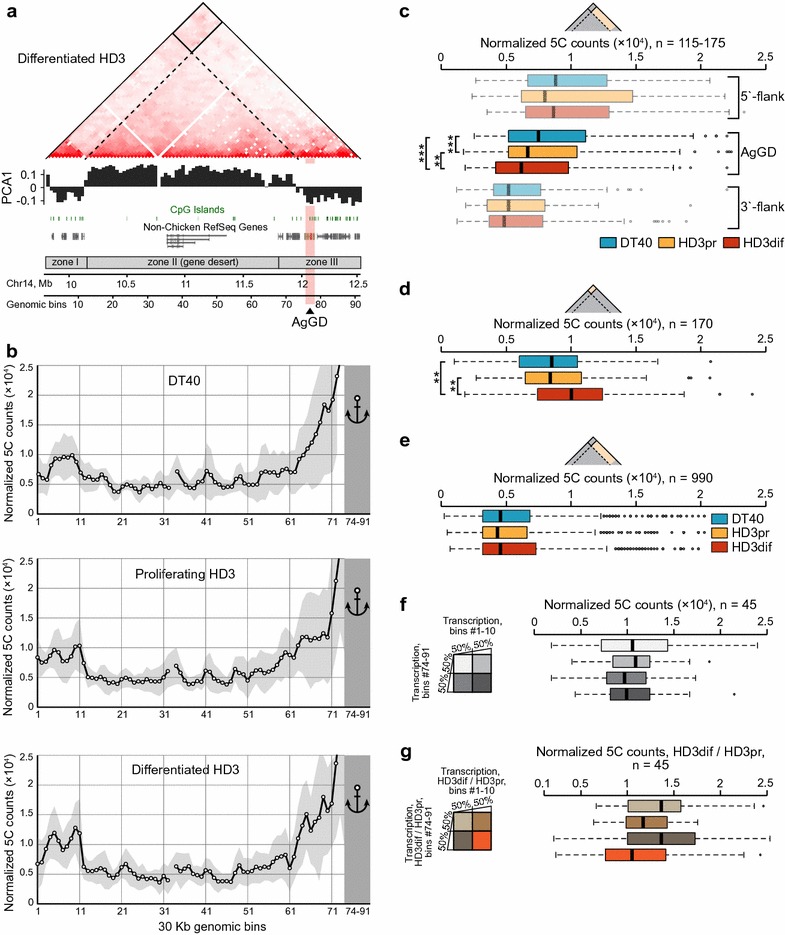
Analysis of A/B-like chromatin compartment structure of the studied genomic region. **a** Heatmap of the differentiated HD3 cells demonstrating an increased interaction frequency between gene-rich zones I and III. A-like and B-like chromatin compartments are outlined using *black rectangle* and *black stipple triangle*, respectively. The first principal component value is shown *below the heatmap*. Structural zones I (5′-terminal gene-rich area), II (gene desert), and III (3′-terminal gene-rich area) were selected manually based on the density of CpG-islands and annotated genes. **b** The averaged interaction profiles of 18 genomic bins #74-91 from zone III (virtual “anchor”) with all bins from the remaining part of the studied region. *Circles* show average values of 5C counts between genomic bins #74-91 with each of the other bins in the studied region; standard deviation is shown. **c** 5C counts corresponding to interactions of the AgGD and flanking regions from the TAD T3 with the gene desert. *Thick black lines* represent median values. *Two asterisks* represent a significant difference with a *P*-value <0.01 calculated using a one-tailed Wilcoxon’s signed-rank test. *Three asterisks* represent a significant difference with a *P*-value <0.001. The same notations are in panels (**d**) and (**e**). **d** The distributions of 5C counts within the A-like chromatin compartment. **e** 5C counts between genomic bins #74-91 and the remaining part of zone III and the gene desert. **f** 5C counts between four groups of bins from zones I and III (differentiated HD3 cells); bins were divided into groups according to their transcription level. **g** 5C counts between four groups of bins from zones I and III; bins were divided into groups according to their degree of the transcription level increase in the HD3dif cells as compared to the HD3pr cells

The 5C heatmaps show non-uniform chromatin compaction along the studied genomic region. A preliminary visual inspection of the heatmaps reveals that the major part of zone III harboring the AgGD interacts more intensively with zone I than with the gene desert (Fig. 2a; Additional file 5: Figure S3a). In mammals, active and repressed genomic regions are spatially segregated into two compartments, namely compartment A containing active genes, and compartment B harboring silent genes and gene deserts [37, 38]. Using principal component analysis [37], we annotated two compartment borders within the studied region (Fig. 2a, see “Methods”). Remarkably, the first compartment border is located exactly at the border between the highly transcribed gene-reach zone I and the gene desert, and coincides with the right boundary of the 5′-terminal TAD (genomic bin #11; see the next section for the description of TAD annotation). The second compartment border occurs at the genomic bin #73 approximately 60 Kb upstream of AgGD. To validate this observation, we constructed a plot featuring the average interaction profile of bins #74-91 located in zone III (a virtual “anchor”) with all bins located upstream of this region. Indeed, the anchor region interacts with zone I more frequently than with the gene desert in all three cell types (Fig. 2b). Notably, a high interaction frequency between the gene-dense zones of the studied region and their spatial separation from the gene desert is supported by our previously published results of 4C experiments with an anchor primer placed at the *NPRL3* promoter [39] (Additional file 5: Figure S3b). Taken together, these observations indicate that the studied region is partitioned into two chromatin compartments in both lymphoid and erythroid cells. The first compartment (negative values of the first principal component, Fig. 2a) consists of the zone I and the larger part of the zone III, which are gene-dense, contain many actively transcribed genes and intergenic regions, and are enriched with CpG-islands. We classified this compartment as A-like. The second compartment (positive values of the first principal component, Fig. 2a) is depleted in CpG-islands, and includes the gene desert and the poorly transcribed 5′-segment of the zone III, and thus may be considered as B-like.

Being located within the zone III, the AgGD belongs to the A-like chromatin compartment in both lymphoid and erythroid cells. To find out whether full activation of the AgGD affects its interaction frequency with the transcriptionally silent gene desert, we compared interaction frequency of the gene desert with the AgGD and with two control 60 Kb regions flanking the AgGD. Despite control regions being transcriptionally upregulated in differentiated HD3 cells along with globin genes (2.6- and 1.7-fold, respectively, as compared to proliferating cells), we observed a significant decrease in the interaction frequency with the gene desert exclusively for the AgGD (*P* < 0.01, one-tailed Wilcoxon’s signed-rank test, Fig. 2c). Thus, transcription activation leads to further segregation of the AgGD from the gene desert.

Upregulation of transcription in zones I and III in differentiated HD3 cells is accompanied by a significant increase in interaction frequency between these two zones belonging to A-compartment (*P* < 0.01, one-tailed Wilcoxon’s signed-rank test, Fig. 2d). Importantly, this fact cannot be explained by the general chromatin compaction during erythroid cell maturation, as the interaction frequency between the zone III and the gene desert is not increased (Fig. 2e). Based on these observations, one may propose that the internal structure of the A-like compartment is affected by the transcription level of the encompassed genes. However, analyzing pairwise interactions of the genomic bins within A-like compartment, we did not find any dependency of the interaction frequency on the level of transcription within the bins (see data for the differentiated HD3 cells in Fig. 2f). Moreover, we observe that the bins characterized by the highest increase of transcription upon HD3 differentiation and show the slightest (if any) increase in contact frequency with each other (orange boxplot in Fig. 2g). Consequently, augmentation of the contact frequency within the A-like compartment and increase in the transcription within zones I and III appear to be concomitant but not causatively related events during HD3 cell differentiation.

Finally, to visualize the overall 3D configuration of the studied region, we performed IMP polymer simulations [40]. The obtained 3D structures (Additional file 6: Figure S4) demonstrate that, in both lymphoid and erythroid cells, the studied region adopts a loop configuration allowing for juxtaposition of gene-rich zones I and III. Terminal differentiation of HD3 cells correlates with compression of the loop, resulting in an increase in interactions between these zones.

### Full activation of alpha-globin gene transcription triggers compaction of the encompassing sub-TAD

**Fig. 3:**
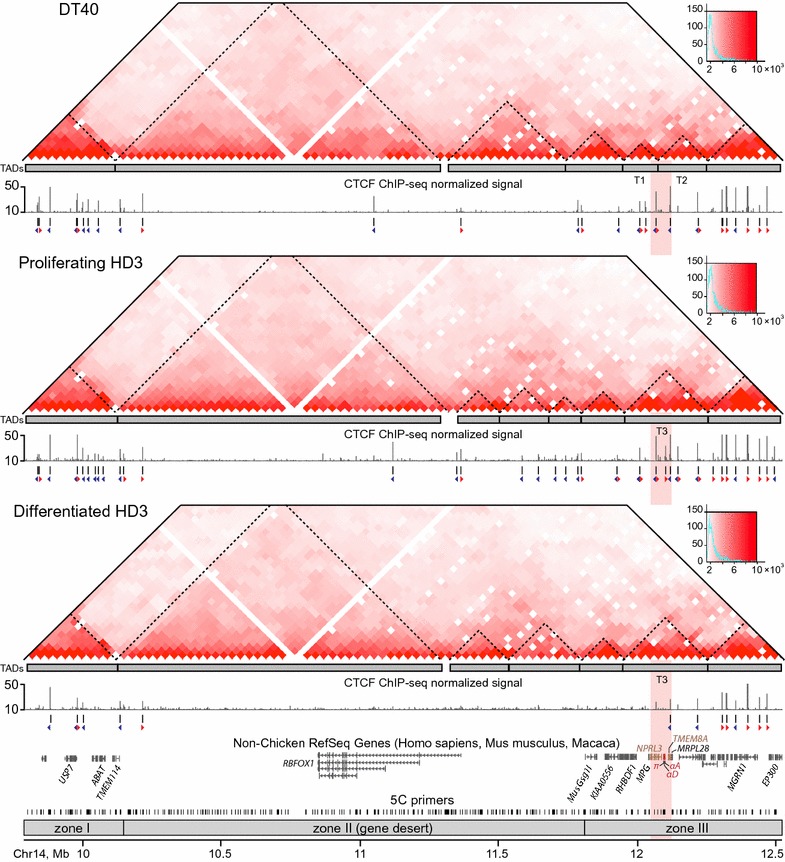
The studied genomic region is partitioned into TADs largely conserved between lymphoid and erythroid cells. The *heatmaps* show 5C data normalized by the total number of sequencing reads in the 5C dataset, binned at a 30 Kb resolution, iteratively corrected and smoothed. Histograms of the 5C counts are shown to the right of the heatmaps. *Gray rectangles below the heatmaps* show TADs that were annotated using the Lavaburst package. TADs T1 and T2 (recognized as fused into one domain T3 in erythroid cells) harbor the alpha-globin gene domain and flanking regions. The graphs demonstrating CTCF binding in the three cell types are based on previously published ChIP-seq data [39]. The direction of forward- and reverse-oriented CTCF binding motifs within the ChIP-seq peaks is shown below the ChIP-seq peaks using *red and blue tailless arrows*, respectively

In all three cell types, self-interacting chromatin domains are clearly visible in the studied region (Fig. 3). To accurately annotate positions of TADs, we used a dynamic programming segmentation algorithm [41] implemented in the Lavaburst package (see “Methods”). Partitioning of the studied region into TADs appears to be robust within a wide range of the parameter settings and largely conserved across the cell types (Additional file 7: Table S3; gamma-value 0.15 was used for the TAD boundary annotation in all cell types). Of note, our data provide the first evidence on the existence of TADs in the chicken interphase chromatin.

The comparison of the TAD positions with the previously reported CTCF ChIP-seq profiles [39] demonstrates high occupancy of CTCF binding sites (CBSs) within TADs located in gene-rich zones I and III in DT40 and proliferating HD3 cells. Notably, most of annotated CBSs in the 5′-terminal TAD contain reverse CTCF binding motifs (reverse CBSs; blue tailless arrows in Fig. 3), the majority of CBSs in the 3′-terminal TAD contain forward motifs (forward CBSs; red tailless arrows in Fig. 3), whereas the area containing AgGD and flanks predominantly contains both forward and reverse CBSs which are always divergently oriented. Terminal differentiation of the HD3 cells is accompanied by a proliferation arrest and genome-wide depletion of CTCF binding [39]. Within the region of interest, there is an almost complete loss of CTCF ChIP-seq peaks within zone III and the gene desert. A partial loss of CTCF binding was observed inside the 3′- and 5′-terminal TADs (Fig. 3). Surprisingly, the decrease in CTCF binding sites occupancy does not lead to dramatic changes in the TAD profile: twelve out of fourteen TAD boundaries identified in proliferating HD3 cells are present in the differentiated cells (Fig. 3; Additional file 7: Table S3). Thus, there should be a mechanism preserving TAD structure in post-mitotic cells even when the CTCF binding is abrogated or substantially depleted.

**Fig. 4:**
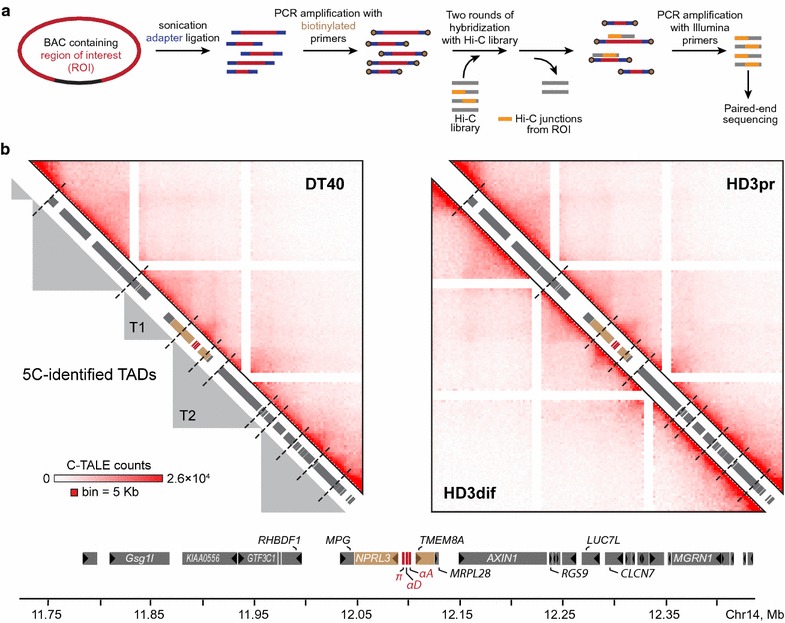
High-resolution C-TALE-identified TAD profile around AgGD. **a** A schematic representation of the C-TALE procedure. For the experimental details, see “Methods” and Additional file 8: Supplementary Methods. **b** Heatmaps showing C-TALE data normalized by the total number of sequencing reads in the C-TALE dataset, binned at a 5 Kb resolution and iteratively corrected. *Dashed lines* show the positions of chromatin contact domain boundaries identified using the Lavaburst package. *Gray triangles in the left panel* represent 5C-identified TADs in DT40 cell line. Gene positions are shown on the diagonal of each panel, and a detailed map of the studied region is presented *below the heatmaps*. *Dark triangles inside the gene boxes* show the direction of transcription

**Fig. 5:**
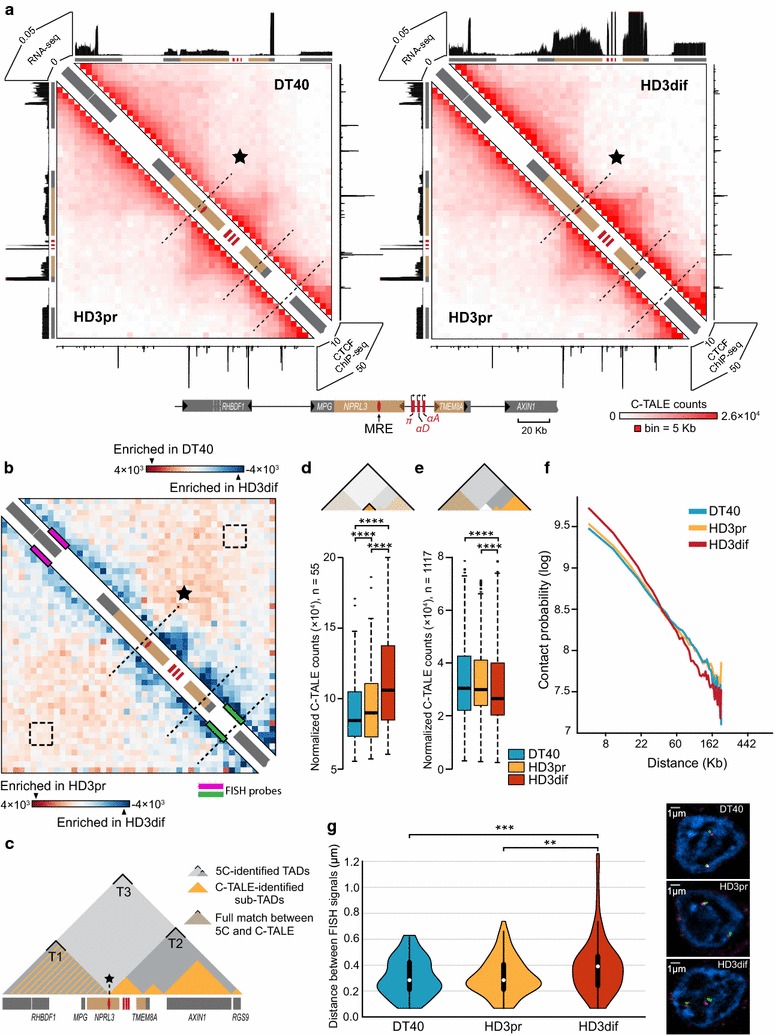
Activation of alpha-globin gene transcription is accompanied by local changes in chromatin interaction profile. **a** C-TALE heatmaps showing local chromatin interaction profile in a close vicinity to AgGD (region chr14:11957600-12236390). *Black star* denotes the boundary between T1 and T2 TADs. All other notations are as in Fig. 4b. **b** C-TALE heatmap showing interactions enriched in differentiated HD3 cells as compared to DT40 cells (right part of the map) and to proliferating HD3 cells (*left part of the map*). The positions of FISH probes are shown with *magenta and green rectangles*. Regions of the map corresponding to interactions between FISH probes are encircled with *dashed squares*. **c** Schematic representation C-TALE-identified sub-TADs within T1 and T2 TADs. **d** The distributions of C-TALE counts corresponding to chromatin interactions inside the sub-TAD harboring alpha-globin gene cluster. *Thick black lines* represent median values. *Asterisks* represent a significant difference with a *P*-value <10^−4^ (one-tailed Wilcoxon’s signed-rank test). **e** The distributions of C-TALE counts corresponding to chromatin interactions between genomic bins, separated by the distance more than 60 Kb throughout the shown region without including the sub-TAD harboring alpha-globin gene cluster. All notations are as in panel (**d**). **f** Scale-plots showing the dependency of contact probability on genomic distance within the shown region. **g** *Violin plots* showing the distributions of spatial distance between FISH probes. *White dots* represent the medians. *Asterisks* represent a significant difference with a *P*-value <0.01 (*two asterisks*), and with a *P*-value <0.001 (*three asterisks*) (Mann–Whitney rank-sum test). Representative examples of FISH images are shown on the *right*. *Scale bar* 1 µm

In lymphoid DT40 cells, the area containing the AgGD and several neighboring genes (from the *RHBDF1* to the *RGS9)* is partitioned into two TADs that are arbitrary designated as T1 and T2 (Fig. 3). In proliferating erythroid HD3 cells, the boundary between these TADs is not annotated. The self-interacting domains T1 and T2 appeared fused into a large loose TAD T3 (Fig. 3). To examine the TAD profile around AgGD more in details, we have developed and applied C-TALE (Chromatin TArget Ligation Enrichment)—a novel cost-effective derivative of the Capture Hi-C technique [42, 43] (Fig. 4a). C-TALE procedure is based on the hybridization of a standard Hi-C library with biotin-labeled fragments of bacterial artificial chromosomes covering the locus of interest with subsequent biotin pull-down and deep sequencing of the trapped Hi-C junctions (see “Methods”, Additional file 8: Supplementary Methods and Additional file 9: Table S5). C-TALE maps for a 735-Kb region centered at the AgGD show that this region is partitioned into eight clearly defined contact domains (CD), and their positions are completely conserved among the three cell types examined (Fig. 4b). Importantly, the boundaries of T1 and T2 TADs perfectly align with the boundaries of C-TALE-identified CDs (Figs. 4b, 5c). At the same time, in contrast to relatively low-resolution 5C data, high-resolution C-TALE analysis does not reveal measurable fusion of T1 and T2 TADs in HD3 cells. C-TALE analysis shows that AgGD-containing T2 TAD has a hierarchical structure and is comprised of four smaller CDs that can be considered as sub-TADs. In the three cell types studied, all genomic elements involved in the formation of the AgGD active chromatin microcompartment (the major enhancer MRE, the *NPRL3*-promoter, the alpha-globin genes, the 3′-enhancer of AgGD and the proximal part of the *TMEM8A* gene containing an erythroid-specific enhancer [28]) are located within a distinct sub-TAD (Fig. 5a). Activation of the alpha-globin gene transcription and the formation of loops between AgGD functional elements [26] are accompanied by a significant compaction of this sub-TAD in differentiated HD3 cells (*P* < 10^−4^, one-tailed Wilcoxon’s signed-rank test, Fig. 5b, d). Along with it, upregulation of six out of seven non-globin genes located within T1 and T2 TADs and almost complete loss of CTCF binding within these domains are accompanied by a remarkable depletion of middle- and long-range chromatin contacts within T1 and T2 (Fig. 5b, e, f), indicating a partial decompaction of an extended 300-Kb region around the alpha-globin gene cluster. To validate this observation, we performed 3D FISH with probes placed within T1 and T2 TADs and separated by 165 Kb (Fig. 5g). In agreement with the results of the C-TALE analysis, we found that in differentiated HD3 cells the probes are separated from each other by a significantly larger distance than in DT40 (*P* = 4.3 × 10^−4^, Mann–Whitney rank test) and in proliferating HD3 cells (*P* = 8.7 × 10^−3^, Mann–Whitney rank test).

### Alpha-globin MRE is involved in long-range looping interactions with CBSs outside of the alpha-globin locus

**Fig. 6:**
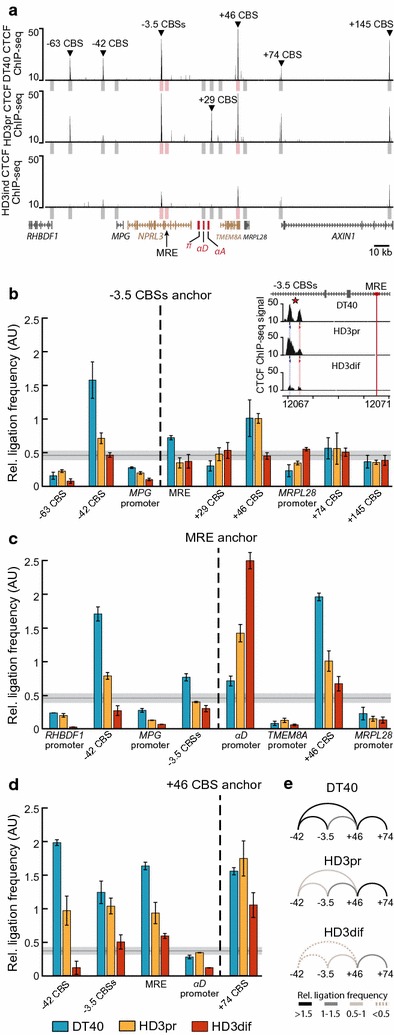
DpnII 3C-analysis of chromatin contacts of the alpha-globin MRE and nearby CTCF-occupied region −3.5 CBSs. **a** Map showing the positions of genes and CTCF ChIP-seq peaks. Test and anchor regions are outlined with *gray and pink vertical rectangles*, respectively. CBSs are designated according to their distance (in kilobases) from the MRE. **b** 3C interaction profile of the −3.5 CBSs. Ligation frequencies averaged between biological replicates are shown. *Error bars* represent SEM. Anchor position relative to positions of test regions is outlined with *vertical dashed line*. *Horizontal gray line* represent a relative noise level ±SEM (see “Methods”). A closer view of the CTCF ChIP-seq profile of −3.5 CBS is shown above the diagram. **c** 3C interaction profile of the MRE. All notations are as in panel (**b**). **d** 3C interaction profile of the +46 CBS. All notations are as in panel (**b**). **e** Schematic representation of the CTCF-anchored loops observed around AgGD

In the three cell types studied, the strong erythroid-specific enhancer of AgGD known as the major regulatory element (MRE) [44] is located in an immediate vicinity of the T1–T2 boundary (Fig. 5a). In DT40 cells, this boundary contains two closely positioned divergent CBSs located 3.5 Kb upstream of the MRE (designated as “−3.5 CBSs”; see Fig. 6a). In proliferating HD3 cells, we observed an approximately twofold decrease in CTCF binding at the forward motif within the −3.5 CBSs, and both peaks are strongly reduced in differentiated HD3 cells (Fig. 6b). Considering that in DT40 and proliferating HD3 cells T1 and T2 TADs harbor multiple CBSs containing binding motifs in both orientations and that the majority of them are lost in differentiated HD3 cells, we assumed that the T1–T2 boundary could be involved in multiple cell type-specific looping interactions in DT40 and proliferating HD3 cells, but not in differentiated HD3 cells. To test this hypothesis, we performed a high-resolution 3C analysis with the anchor primers placed in DpnII restriction fragments harboring MRE and the −3.5 CBSs.

As anchor primers at −3.5 CBSs and at MRE are located close to each other, we expected that their 3C profiles would be similar. Indeed, in DT40 cells, both anchors interact strongly with the forward-orientated −42 CBS located 2 Kb upstream of the *MPG* gene (Fig. 6b, c). In both proliferating and differentiated HD3 cells (consistently with the depletion of CTCF binding at the −42 CBS), we observed a two- to threefold depletion of this interaction with both anchors. In a downstream direction relative to the anchor positions, in DT40 and proliferating HD3 cells both anchors exhibit strong looping with the reverse-oriented +46 CBS located within the *TMEM8A* gene. Unexpectedly, in lymphoid cells, the MRE interacts with the +46 CBS much stronger than the −3.5 CBSs, and that was confirmed in a reciprocal experiment with the anchor primer placed at the +46 CBS (Fig. 6d). Moreover, in proliferating HD3 cells, the interaction of the +46 CBS with the MRE considerably decreases, whereas the interaction with the −3.5 CBSs does not change. Taken together, these observations suggest that the MRE potentially forms a direct loop with the +46 CBS in both lymphoid and erythroid cells. Notably, the MRE/+ 46 CBS looping appears to be an alternative for the formation of the alpha-globin active chromatin hub, because our 3C analysis does not reveal reliable spatial interaction between the +46 CBS and the *α* ^*D*^ promoter in any cell type.

Additionally, our 3C analysis does not show spatial contacts between the MRE and the promoters of non-globin genes *RHBDF1*, *MPG*, *TMEM8A,* and *MRPL28* flanking the alpha-globin gene locus (Fig. 6c). Thus, upregulation of the three former genes and downregulation of the *MRPL28* gene in differentiated HD3 cells is not related to looping interactions with MRE.

## Discussion

Alpha-globin gene domains of warm-blooded animals are located within areas enriched in widely expressed genes [15, 45]. Here, we find that the majority of such genes surrounding the chicken alpha-globin cluster are substantially upregulated in erythroid cells induced to terminal differentiation (Fig. 1b, c; Additional file 2: Table S1). At first glance, such upregulation could be considered generally erythroid-specific, particularly for the *TMEM8A* that is slightly upregulated even in proliferating HD3 cells as compared to DT40 cells (Fig. 1c, left plot). However, in the cell line K562, a human counterpart of the chicken HD3 cells, induction of differentiation leads to upregulation of the *NPRL3* gene but not *TMEM8A* [28, 46] which in the human genome is located in the neighboring TAD 200 Kb downstream of the AgGD [4]. Consistently, it has been previously suggested that upregulation of the chicken *TMEM8A* gene in differentiated erythroid cells is likely to be a consequence of it being in close proximity to AgGD enabling looping interactions between *TMEM8A* and alpha-globin-specific enhancers (−9DHS and 3′-enhancer) but not with the MRE [28] (the latter is supported by our data, Fig. 6c).

In contrast, the genomic position of *NPRL3* relative to the alpha-globin gene cluster and its involvement in the regulatory and spatial network of AgGD is conserved in chicken and mammals [18, 26]. The *NPRL3* promoter interacts with the MRE, and that could potentially cause its upregulation in differentiated erythroid cells, which, in turn, could have some function in the erythropoiesis. Although there are no studies of the role for the *NPRL3* protein product in the maturation of red blood cells, it has been shown that early defects of hematopoiesis in mice may be caused by the knock-out of the NPRL2, a paralog [47] and interacting partner of the NPRL3 within the GATOR1 complex regulating the mTOR pathway [48]. Moreover, *NPRL3* is transcribed at high level during normal in vitro differentiation of the primary human erythroid cells [20, 49]. Thus, transcriptional upregulation of the widely expressed gene *NPRL3* in differentiated erythroid cells is evolutionarily conserved across mammals and birds, and we propose that it might be functionally related to the definitive erythropoiesis in homeotherms.

Similarly to the *NPRL3*, genomic location of two more housekeeping genes *RHBDF1* and *MPG* upstream of the *NPRL3* is highly conserved in evolution of vertebrates [50]. Upregulation of these genes in differentiated HD3 cells (Additional file 2: Table S1) could be explained by some indirect mechanisms of stimulation rather than by immediate impact of MRE, as our 3C analysis shows that MRE does not spatially interact with their promoters (Fig. 6c). In this regard, abundant intergenic transcription covering the entire AgGD in differentiated HD3 cells might be of particular interest. It has been proposed that intergenic transcription promotes activation of genomic domains via facilitated spreading of histone acetyltransferases and chromatin remodeling complexes bound to the C-terminal tail of the elongating RNA polymerase II [51]. Possibly, this intergenic transcription contributes to the chromatin decompaction around AgGD (Fig. 5b) in differentiated HD3 cells that induces upregulation of adjacent genes via the increased chromatin accessibility to transcription factors.

At the same time, activation of alpha-globin gene transcription is accompanied by an extensive increase in chromatin interactions within the encompassing sub-TAD (Fig. 5b, d). In chicken erythroid cells, the alpha-globin locus is assembled into an active chromatin hub (ACH) [26]. Formation of this ACH comprised of several looping interactions between functional elements of the locus may explain the overall chromatin compaction within the entire sub-TAD. One of the first steps in the assembly of the alpha-globin ACH is the establishment of spatial contacts between the MRE, the *NPRL3* promoter, and the promoter of the *α* ^*D*^ gene [26]. The results of our experiments suggest that, to participate in the assembly of ACH, the MRE should be first released from its interactions with the upstream (−42) and the downstream (+46) CBSs. It is tempting to speculate that the removal of CTCF from the T1–T2 boundary and weakening of the corresponding loops (Fig. 6a, e) triggers a spatial reconfiguration of the domain, allowing for the assembly of the alpha-globin ACH and reconfiguration of the encompassing sub-TAD. The spectrum of spatial contacts of the MRE in the lymphoid cells (and partially in proliferating HD3 cells) could be constrained by strong interactions of the upstream −3.5 CBS (Fig. 6b). In other words, when the −3.5 CBS located close to MRE is occupied by CTCF, MRE could preferentially contact the interaction partners of this CBS outside of AgGD. Such looping would restrict the possibility of establishing spatial contacts between the MRE and the downstream promoters of alpha-globin genes.

In differentiated HD3 cells, an almost complete loss of the CTCF binding is observed throughout the AgGD neighborhoods (Figs. 3, 5a). On the one hand, it reflects a general decrease in CTCF binding genome-wide—8768 and 4820 CBSs are detected in proliferating and differentiated HD3 cells, respectively (GSE51939 [39]). On the other hand, attenuation of CTCF binding within the T1 and T2 TADs is much more drastic than within two other gene-rich regions of the studied chromosome segment (5′- and 3′-terminal TADs) (Fig. 3). Numerous studies performed in human and murine cultured and primary cell lines have identified CTCF as a key regulator of TAD formation in mammals [4, 52–54]. However, we do not see the loss of TADs or dramatic changes in the TAD profile in differentiated HD3 cells in spite of dramatic loss of CTCF occupancy at the binding sites within the studied area (Fig. 3; Additional file 7: Table S3). Importantly, using the 3C analysis we demonstrate that the strength of all CTCF-anchored loops detected within the T1 and T2 TADs is considerably decreased in differentiated HD3 cells (Fig. 6e). Surprisingly, this does not lead to obvious changes in the sub-TAD profile in this region. These results are in agreement with recently reported observations, suggesting that induced degradation of CTCF in mouse embryonic stem cells does not largely affect the positions of TAD boundaries [55]. Thus, some other additional mechanisms may be responsible for the maintenance of preformed TADs when the CTCF binding at CBSs is decreased. One possibility is that the tertiary structure of the chromatin fiber (self-interacting globule) that is initially established by the CTCF/cohesin-dependent looping is further stabilized and maintained by interactions between nucleosomes based on the mechanism of TAD formation in the *Drosophila* genome that we recently proposed [56]. To this end, it may be of importance that terminally differentiated HD3 cells do not divide. Consequently, the higher-order chromatin structure is not perturbed by mitosis.

In this study, we demonstrated for the first time the presence of TADs and A- and B-like compartments in chicken genome. Although the profiles of A- and B-like chromatin compartments within the studied genomic segment are similar if not identical in the three cell types studied, the contact frequency within the A-like compartment increases in differentiated HD3 cells (Fig. 2d). Surprisingly, we did not find any relationship between the increase in 5C counts and the increase in of transcription within the respective genomic bins (Fig. 2f, g). This observation argues that the nature of long-range interactions within chromatin compartments could be predominantly stochastic and determined by co-occurrence of chromatin domains within the same volume of nuclear space rather than by specific associations of highly transcribed genes. Consistently with the previously observed escape of upregulated genes from the B-compartment during human ESC differentiation [57], full activation of the alpha-globin transcription in differentiated HD3 cells is accompanied by a further segregation of the AgGD from the gene desert (Fig. 2c). Interestingly, other regions of the T1 and T2 TADs do not demonstrate changes in contact frequency with the gene desert, showing that different parts of the same TAD may possess a certain degree of autonomy in establishing long-range spatial contacts.

## Conclusions

Taken together, our findings suggest that full activation of the chicken alpha-globin gene domain in differentiated erythroid cells correlates with local as well as large-scale changes in the chromatin folding and gene expression within an extended chromosome region. It contradicts simple models of the genome partitioning into functionally isolated gene domains sequentially located along the chromosome and argues that the effects of tissue-specific transcription activation are not restricted to the host genomic locus but affect the overall chromatin structure and transcriptional output of an extended genomic neighbourhood. At the same time, our observations demonstrate that local reconfiguration of a tissue-specific gene domain may be guided by changes in the 3D packaging of an extended genomic region.

## Methods

### Cell culture

The avian erythroblastosis virus-transformed chicken erythroblast cell line HD3 (clone A6 of line LSCC) and the chicken lymphoid cell line DT40 (CRL-2111, ATCC, London, UK) were grown in Dulbecco’s modified Eagle’s medium (DMEM) supplemented with 2% chicken serum and 8% fetal bovine serum at 37 °C in 5% CO_2_ atmosphere. To induce terminal erythroid differentiation, HD3 cells (8 × 10^5^ cells/ml) were incubated in the full growth media supplemented with 10 mM HEPES (pH 8.0) and 20 μM iso-H-7 (1-(5-isoquinolinylsulfonyl)-3-methylpiperazine dihydrochloride, Fluka, Seelze, Germany) at 42 °C in 100% air atmosphere.

### 3C library preparation

3C libraries were prepared as described previously [58] with minor modifications. Briefly, after fixation with 2% formaldehyde, the cells were lysed for 15 min in 1.5 ml of ice-cold isotonic lysis buffer (50 mM Tris–HCl (pH8.0), 150 mM NaCl, 0.5% (v/v) NP-40, 1% (v/v) Triton X-100 and 1× Protease Inhibitor Cocktail (Thermo Scientific #78430)). The nuclei were harvested, washed twice with 1.12× restriction enzyme buffer (NEB), and then treated with 0.3% SDS which was subsequently diluted to a concentration of 0.1% with 1.12× restriction enzyme buffer and quenched with 1.8% Triton X-100. DNA was digested overnight with 600 U of 100 U/μl of HindIII-HF restriction endonuclease (NEB) or 600 U of 50 U/μl of DpnII restriction endonuclease (NEB) at 37 °C with shaking. The next morning, 200 U of the corresponding restriction enzyme was added, and the samples were incubated for 2 h under the same conditions. Then, the samples were heated to 65 °C for 20 min to inactivate the restriction enzyme. The nuclei were washed twice with 100 μl of 1× T4 DNA ligase reaction buffer (Thermo Scientific) and resuspended in 300 μl of 1× T4 DNA ligase reaction buffer, 75 U of T4 DNA ligase (Thermo Scientific) was added, and the DNA was ligated for 6 h at 16 °C with constant shaking (1400 rpm). After cross-link reversal, RNAse A treatment, and single phenol–chloroform extraction followed by ethanol precipitation, the DNA was additionally purified using AMICON Ultra Centrifugal Filter Units (0.5 ml, 30 K, Millipore #UFC5030BK) to remove residual salts and DTT.

For the detection of the ligation products in DpnII-3C libraries, real-time PCR with TaqMan probes was employed. Primers and TaqMan probes for PCR analysis were designed using Primer Premier 5 software (PRIMER Biosoft International). The sequences of the primers and TaqMan probes are present in Additional file 10: Table S4. A total of 400 ng of the DpnII-3C library was used as a template in PCR. Calibration curves were constructed using four tenfold dilutions of the BAC standard template (200, 20, 2, and 0.2 pg of the BAC template supplemented with 50 ng of the chicken genomic DNA per PCR reaction) prepared from an equimolar mixture of the bacterial artificial chromosomes CH261-75C12 and CH261-85E12 (CHORI BACPAC). All 3C experiments were carried out in at least two independent biological replicates. Relative noise level in 3C plots was calculated as described in [59].

### 5C primer design

5C primers covering region 9.8–12.5 Mb of chicken chromosome 14 were designed for HindIII restriction fragments using the alternating scheme in my5C.primers [60] with the following parameters: (1) an optimal primer length of 30 bp; (2) an optimal Tm of 65 °C; and (3) a default primer quality parameters (mer: 800, U-blast: 3 and S-blast: 50). Primers were not designed for large (>50 Kb) and small (<100 bp) restriction fragments, low-complexity and repetitive sequences. Primers with sequence matches to more than one genomic target were discarded.

The universal T7-tail (5′-TAATACGACTCACTATAGCC-specific part-3′) and T3-tail (5′-TCCCTTTAGTGAGGGTTAATA-specific part-3′) were added to the forward and reverse 5C primers, respectively. 5′-ends of reverse 5C primers were phosphorylated. In total, 93 forward and 92 reverse primers were designed (Additional file 4: Table S2).

### 5C library preparation

5C libraries were prepared in two biological replicates as described previously [36] with minor modifications. Briefly, 5C primer stocks (17 mM) were mixed and diluted in water to a final concentration of 1.7 nM of each primer. 5C primers were mixed with 3C libraries in 10 μl of annealing mixtures of the following composition: 1 μl of 10× NEBuffer 4 (NEB), 1.7 μl of 5C primer mix, 600 ng of 3C library, 900 ng of salmon testis DNA (Sigma) sonicated to a size of 100–500 bp, and mQ water. Samples were denatured for 5 min at 95 °C and annealed for 16 h at 57 °C. Samples were supplemented with pre-heated 1× Taq DNA ligase buffer, and ligation with 10 U of Taq DNA ligase (NEB) was performed for 1 h at 57 °C. After that, the samples were quickly frozen at −80 °C. A total of 2 μl of each 5C library was then PCR-amplified individually with primers to T7- and T3-tails using KAPA High Fidelity DNA Polymerase (KAPA). The temperature profile was 5 min at 98 °C, followed by 22, 24, 26, 28, and 30 cycles of 20 s at 98 °C, 15 s at 65 °C, and 20 s at 72 °C. The PCR reactions were separated on a 2% agarose gel supplied with ethidium bromide, and the number of PCR cycles necessary to obtain a sufficient amount of DNA was determined based on visual inspection of gels (typically 28–30 cycles). Four preparative PCR reactions were performed for each sample. The PCR mixtures were combined, and the products were purified with a QIAGEN PCR Purification Kit. The DNA was eluted with 50 μl of 10-mM Tris–HCl (pH 8.0) and separated on a 1.8% agarose gel supplied with ethidium bromide; 100-bp fragments were excised from the gel, purified using a QIAGEN Gel Extraction Kit and sequenced on Illumina HiSeq 2000 using single-end 101-nt reads.

### C-TALE library preparation

For the experimental details, see Additional file 8: Supplementary Methods. Briefly, biotinylated hybridization baits were produced from BACs covering the region of chromosome 14 with coordinates 11721574-12436767 (galGal4 genome assembly) by ultrasonic fragmentation of BAC DNA to a size of 200–600 bp with subsequent adapter ligation and PCR amplification with biotinylated primers. Hi-C libraries were hybridized with the baits for 40 h at 65 °C, the trapped portion of the Hi-C libraries was PCR-amplified with Illumina PE1.0 and PE2.0 primers, and the products of amplification were subjected to the second round of hybridization with the same conditions. After that, trapped fragments of the Hi-C library were PCR-amplified with Illumina PE1.0 and PE2.0 primers and sequenced on Illumina HiSeq 2000 using paired-end 101-nt reads.

### 5C raw data processing and analysis, and CTCF binding motif search

Each 5C experiment was performed in two biological replicates (Additional file 3: Figure S2). 5C reads were checked for the presence of the HindIII restriction site and divided into two groups corresponding to forward and reverse primers in an interacting pair of primers. These read groups were mapped independently with bowtie2 [61] to the studied region of chicken chromosome 14 (genome assembly galGal4). To filter out low-coverage data, we excluded all primers with zero interactions in at least one cell type. The filtered data were binned in 30 Kb windows and the mean 5C count per bin was calculated, resulting in a matrix of interactions between forward and reverse primers. Bins corresponding to interactions within the same fragment were removed. We then applied the iterative correction procedure [38] to reduce intrinsic bin-specific biases. Because biological replicates demonstrated high correlation for all three cell types (Pearson’s correlation coefficient >0.9, Additional file 3: Figure S2), we merged them by summing their matrices element by element. To reduce noise and achieve better visualization, we smoothed the data by assigning each matrix element the median 5C count between this element and its adjacent elements. Zero matrix elements were not smoothed to avoid inflating low-confidence interaction values.

TADs were identified using the Lavaburst package (https://github.com/mirnylab/lavaburst), which provides a set of dynamic programming algorithms to assess an ensemble of TAD segmentations derived from a TAD scoring function. We used its optimal segmentation finder, which is based on the Armatus algorithm [41] using the TAD scoring function from that study. The algorithm finds the global TAD segmentation of a contact map having the highest aggregate score.

Chromatin compartments were identified using the principal component analysis, as described previously [37].

A CTCF motif search was performed using the Biopython motif search method [62] and the human CTCF position-frequency matrix from the JASPAR database [63].

### C-TALE raw data processing and analysis

Illumina HiSeq 2500 bcl2fastq v1.8.4 Conversion Software was used for base-calling. The reads were mapped independently to the region 11700000-12450000 of chromosome 14 using hiclib [38] (https://bitbucket.org/mirnylab/hiclib/) iterative mapping procedure (minimal mapping length 25, iteration step 5) and Bowtie 2 version 2.2.1.

Reads mapped in close proximity to DpnII restriction sites (5 bp) and possible PCR duplicates were eliminated. We also excluded reads mapped to extremely short (<100 bp) and long (>100 Kb) restriction fragments. The reads in pairs that mapped to the same restriction fragment or closer than 500 bp to each other in genomic coordinates were excluded as potential self-ligations and dangling end products. Uniquely mapped read pairs that passed filtering procedure were retained. Resulting pairs were binned into 5 Kb genomic windows of the region of interest. Bins corresponding to interactions within the same fragment were removed. For each bin, the mean C-TALE read count was calculated. To merge C-TALE replicates, we summed their matrices element by element. Then, the matrices were iteratively corrected [38]. TAD annotation was performed with the Lavaburst package as described for 5C. To minimize the effect of missing data on TADs annotation procedure, we removed bins with no interactions from the matrices prior to TAD annotation.

### 3D DNA fluorescence in situ hybridization (3D FISH)

The cells were harvested overnight on poly-l-lysine coated coverslips placed in culture flasks. The cells were fixed in 4% paraformaldehyde for 10 min, permeabilized in 0.5% Triton X-100, washed in PBS, dehydrated in ethanol series, air-dried, stored at room temperature for two days, and then frozen at −80 °C. Probes were PCR-amplified from the CH261-75C12 BAC, primer sequences are presented in Table S4 (Additional file 10). Probes were labeled with biotin or digoxigenin using nick-translation. Approximately 150 ng of each probe was used in hybridization. Denaturation was performed at 80 °C for 30 min in 70% formamide (pH7.5), 2xSSC. Hybridization of probes was done for 24 h in 50% formamide, 2x SSC, 10% dextran sulfate, 1% Tween 20. Washing steps were performed in 2x SSC at 45 °C followed by 0.1x SSC at 60 °C and 4x SSC, 0.1% Triton X-100. For imaging, cells were counterstained with DAPI and epifluorescent images were acquired using a Photometrics Coolsnap HQ2 CCD camera and a Zeiss Imager A1 fluorescence microscope with a Plan Apochromat 100x 1.4NA objective, a Lumen 200-W metal halide light source (Prior Scientific Instruments, Cambridge, UK) and Chroma #89014ET single excitation and emission filters (Chroma Technology Corp., Rockingham, VT) with the excitation and emission filters installed in Prior motorized filter wheels. A piezoelectrically driven objective mount (PIFOC model P-721, Physik Instrumente GmbH & Co, Karlsruhe) was used to control movement in the z dimension. Hardware control, image capture, deconvolution (using the fast algorithm) and analysis were performed using Volocity (PerkinElmer Inc, Waltham, MA).

### Polymer simulations

We used the integrative modeling platform (IMP) [40] to model the 3D-chromatin structure from the 5C data as described previously (24). We assigned a particle to each 30 Kb bin in smoothed 5C matrices. To use all of the information from the 5C experiment, we set parameters uZ and lZ to zeros; the mP parameter was set to 400 nm. A total of 5000 IMP runs were performed, and one thousand best models were used for further analysis. As a measure of the model quality, we used the Spearman’s correlation coefficient between the modeled matrix of interactions and the experimental 5C matrix (Additional file 6: Figure S4). The TADbit program (https://github.com/3DGenomes/tadbit) was used for all calculations. Chimera [64] and PyMOL (The PyMOL Molecular Graphics System, version 1.7.4 Schrodinger, LLC), tools were used to visualize the models. We used the centroid model for the wireframe representation, and the Gaussian approximation for the surface representation.

### Total rRNA-depleted RNA-seq: experimental procedures and analysis

RNA-seq experiments were performed in two independent biological replicates, according to ENCODE recommendations (https://genome.ucsc.edu/ENCODE/protocols/dataStandards/ENCODE_RNAseq_Standards_V1.0.pdf). The two biological replicates demonstrated a high correlation in all three cell types (Pearson’s *r* > 0.95).

Total RNA was extracted from the cells using TRIzol Reagent (Thermo Scientific) according to the manufacturer’s protocol. Total RNA was depleted with ribosomal RNA using the RiboZero Human/Mouse Kit (Illumina). For library preparation, a TruSeq Stranded RNA Sample Prep Kit (Illumina) was used following the manufacturer’s instructions. After preparation, the quality of the libraries was evaluated using quantitative PCR and Bioanalyzer.

Illumina Casava 1.8.2 was used for base-calling. Reads were mapped to the *Gallus gallus* reference genome (version galGal4) using the TopHat program [65] taking the ENSEMBL gene annotation into account (Ensembl Release 82) with the -no-novel-juncs option. The Htseq-counts program [66] was used to calculate per gene counts. The BedTools program [67] was used to calculate read coverage.

We used R package edgeR for gene expression analysis. Only genes with CPM > 1 in at least two out of six RNA-seq experiments were retained. We averaged CPM over replicates and calculated fold change of expression between experiments. *P*-values were calculated per gene using quasi-likelihood negative binomial generalized log-linear model (glmQLFit function in edgeR). The *P*-values were adjusted with Benjamini–Hochberg correction for multiple testing. The threshold for false discovery rate was set to 1e-7 for detection of differentially regulated genes. For gene ontology analysis, we used DAVID as implemented in RDAVIDWebService R package. We assessed the enrichment of gene ontology terms of “Biological Process” category for the genes that changed expression level more than twofold with adjusted *P*-value <1e-04. The minimal number of genes present in the gene ontology category was set to 5. Resulting DAVID *P*-values of enriched terms were adjusted with Bonferroni correction.

To assess the intergenic transcription levels, we retrieved the regions between ENSEMBL genes and calculated read coverage with BedTools software [67]. To compare the transcription level between experiments for each intergenic loci, log2-fold change of coverage was calculated. We obtained the distribution of fold change between experiments for all intergenic regions and assessed the percentile rank of loci within the studied region.

## Additional files

**Additional file 1: Figure S1.** Analysis of the total rRNA-depleted RNA-seq data in the three studied cell types. **(A)** The Venn diagram showing the numbers of active genes shared between the studied cell types. **(B)** Cluster analysis of biological replicates based on total rRNA-depleted RNA-seq data. **(C)** Principal component analysis of the RNA-seq data. **(D)** Top-30 gene ontology terms for the genes downregulated (logFC < −0.6, FDR < 10^−7^) in differentiated HD3 cells compared to proliferating HD3 cells. The number of genes in each term is shown. **(E)** Scatter plots showing normalized level of transcription within intergenic regions genome-wide in the studied cell types.

**Additional file 2: Table S1.** Transcription level of the genes from the studied region (CPM). Only genes with CPM > 1 in at least 2 replicates are presented. CPM values were averaged over replicates. Log2-fold change and FDR of difference between experiments are presented (see “Methods”). For genes that have not passed filtering procedure, only CPM are present.

**Additional file 3: Figure S2.** Biological replicates of the 5C experiment. Color intensity in each cell of the heatmaps represents the interaction frequency of two corresponding forward and reverse 5C primers. Histograms of the interaction counts are shown to the right of the heatmaps.

**Additional file 4: Table S2.** Sequences and characteristics of the 5C primers used in this study. 5C primers were designed using the alternating scheme in my5C.primers and chicken reference genome assembly galGal4.

**Additional file 5: Figure S3.** The chromatin compartment profile along the studied genomic region. **(A)** Heatmaps demonstrating an increased interaction frequency between gene-rich zones I and III in both lymphoid and erythroid cells. A-like and B-like chromatin compartments are outlined using black opened rectangle and black dotted triangle, respectively. The first principal component is shown below the heatmaps. **(B)** The 4C profiles from [39] revealing an increased interaction frequency of the alpha-globin gene domain (zone III of the studied region) with the USP7-ABAT locus (zone I), and the spatial separation of the gene desert from flanking gene-dense areas in proliferating HD3 and DT40 cells. Positions of the anchor primers are shown using red vertical lines; the alpha-globin gene domain is highlighted with a vertical pink rectangle. The first principal component (PCA1) of the 5C data (DT40 cells) is shown below the 4C profiles. The blue rectangle below the genomic coordinates track represents the genomic region analyzed in this work. The scale is changed at the bottom of the panel to emphasize the borders of 4C counts peak around the anchor. Positions of TADs that were identified using the 5C analysis are highlighted using gray rectangles.

**Additional file 6: Figure S4.** **(A)** Interaction heatmaps of the simulated polymer calculated with TADbit. Color bars in the heatmaps represent arbitrary partitioning of the studied region into several distinct fragments. **(B)** IMP-derived 3D models of the studied genome region. The central wireframe colored as the color bar in the map of the studied region represents the centroid model for simulations. The surface represents the Gaussian approximation of 1000 simulated models.

**Additional file 7: Table S3.** Genomic coordinates of topologically associating domains within the studied region. TADs were identified using the optimal segmentation algorithm from the Lavaburst package with the with the Armatus scoring function.

**Additional file 8: Supplementary Methods.** A detailed description of the C-TALE library preparation.

**Additional file 9: Table S5.** C-TALE sequence statistics.

**Additional file 10: Table S4.** Sequences of primers and TaqMan probes used for the 3C analysis. Sequences of primers used for FISH probe amplification.

## Abbreviations

3C: chromosome conformation capture
3D: three-dimensional
5C: chromatin conformation capture carbon copy
ACH: active chromatin hub
AgGD: alpha-globin gene domain
bp: base pair
CBS: CTCF-binding site
ChIP-seq: chromatin immunoprecipitation followed with deep sequencing
C-TALE: Chromosome TArget Ligation Enrichment
ESC: embryonic stem cells
IMP: integrative modeling platform
Kbp: kilobases, thousands of base pairs
Mb: megabases, millions of base pairs
MRE: major regulatory element
PCA: principal component analysis
TAD: topologically associating domain

## References

[1] Ulianov SV, Gavrilov AA, Razin SV (2015). Nuclear compartments, genome folding, and enhancer–promoter communication. Int Rev Cell Mol Biol.

[2] Vernimmen D, Bickmore WA (2015). The hierarchy of transcriptional activation: from enhancer to promoter. Trends Genet.

[3] Dekker J, Mirny L (2016). The 3D genome as moderator of chromosomal communication. Cell.

[4] Rao SS, Huntley MH, Durand NC, Stamenova EK, Bochkov ID, Robinson JT (2014). A 3D map of the human genome at kilobase resolution reveals principles of chromatin looping. Cell.

[5] Mifsud B, Tavares-Cadete F, Young AN, Sugar R, Schoenfelder S, Ferreira L (2015). Mapping long-range promoter contacts in human cells with high-resolution capture Hi-C. Nat Genet.

[6] Remeseiro S, Hornblad A, Spitz F (2015). Gene regulation during development in the light of topologically associating domains. Wiley Interdiscip Rev Dev Biol.

[7] Lupianez DG, Kraft K, Heinrich V, Krawitz P, Brancati F, Klopocki E (2015). Disruptions of topological chromatin domains cause pathogenic rewiring of gene–enhancer interactions. Cell.

[8] Valton AL, Dekker J (2016). TAD disruption as oncogenic driver. Curr Opin Genet Dev.

[9] Symmons O, Uslu VV, Tsujimura T, Ruf S, Nassari S, Schwarzer W (2014). Functional and topological characteristics of mammalian regulatory domains. Genome Res.

[10] Razin SV, Ulianov SV, Ioudinkova ES, Gushchanskaya ES, Gavrilov AA, Iarovaia OV (2012). Domains of alpha- and beta-globin genes in the context of the structural-functional organization of the eukaryotic genome. Biochemistry (Mosc).

[11] Vernimmen D (2014). Uncovering enhancer functions using the alpha-globin locus. PLoS Genet.

[12] Hughes JR, Cheng JF, Ventress N, Prabhakar S, Clark K, Anguita E (2005). Annotation of cis-regulatory elements by identification, subclassification, and functional assessment of multispecies conserved sequences. Proc Natl Acad Sci USA.

[13] Higgs DR, Wood WG, Jarman AP, Sharpe J, Lida J, Pretorius I-M (1990). A major positive regulatory region located far upstream of the human α-globin gene locus. Genes Dev.

[14] Higgs DR, Vernimmen D, Wood B (2008). Long-range regulation of alpha-globin gene expression. Adv Genet.

[15] Craddock CF, Vyas P, Sharpe JA, Ayyub H, Wood WG, Higgs DR (1995). Contrasting effects of alpha and beta globin regulatory elements on chromatin structure may be related to their different chromosomal environments. EMBO J.

[16] Mahajan MC, Karmakar S, Newburger PE, Krause DS, Weissman SM (2009). Dynamics of alpha-globin locus chromatin structure and gene expression during erythroid differentiation of human CD34(+) cells in culture. Exp Hematol.

[17] Anguita E, Johnson CA, Wood WG, Turner BM, Higgs DR (2001). Identification of a conserved erythroid specific domain of histone acetylation across the alpha-globin gene cluster. Proc Natl Acad Sci USA.

[18] Vernimmen D, Marques-Kranc F, Sharpe JA, Sloane-Stanley JA, Wood WG, Wallace HA (2009). Chromosome looping at the human alpha-globin locus is mediated via the major upstream regulatory element (HS -40). Blood.

[19] Vernimmen D, De Gobbi M, Sloane-Stanley JA, Wood WG, Higgs DR (2007). Long-range chromosomal interactions regulate the timing of the transition between poised and active gene expression. EMBO J.

[20] Lower KM, Hughes JR, De Gobbi M, Henderson S, Viprakasit V, Fisher C (2009). Adventitious changes in long-range gene expression caused by polymorphic structural variation and promoter competition. Proc Natl Acad Sci USA.

[21] Bau D, Sanyal A, Lajoie BR, Capriotti E, Byron M, Lawrence JB (2011). The three-dimensional folding of the alpha-globin gene domain reveals formation of chromatin globules. Nat Struct Mol Biol.

[22] Zhou GL, Xin L, Song W, Di LJ, Liu G, Wu XS (2006). Active chromatin hub of the mouse alpha-globin locus forms in a transcription factory of clustered housekeeping genes. Mol Cell Biol.

[23] Therwath A, Mengod G, Scherrer K (1984). Altered globin gene transcription pattern and the presence of a 7–8 kb alpha A globin gene transcript in avian erythroblastosis virus-transformed cells. EMBO J.

[24] Razin SV, Petrov P, Hancock R (1991). Precise localization of the alpha-globin gene cluster within one of the 20- to 300-kilobase DNA fragment released by cleavage of chicken chromosomal DNA at topoisomerase II sites in vivo: evidence that the fragments are DNA loops or domains. Proc Natl Acad Sci USA.

[25] Valdes-Quezada C, Arriaga-Canon C, Fonseca-Guzman Y, Guerrero G, Recillas-Targa F (2013). CTCF demarcates chicken embryonic alpha-globin gene autonomous silencing and contributes to adult stage-specific gene expression. Epigenetics.

[26] Gavrilov AA, Razin SV (2008). Spatial configuration of the chicken α-globin gene domain: immature and active chromatin hubs. Nucleic Acids Res.

[27] Gasaryan KG (1982). Genome activity and gene expression in avian erythroid cells. Int Rev Cytol.

[28] Philonenko ES, Klochkov DB, Borunova VV, Gavrilov AA, Razin SV, Iarovaia OV (2009). TMEM8—a non-globin gene entrapped in the globin web. Nucleic Acids Res.

[29] Broders F, Zahraoui A, Scherrer K (1990). The chicken alpha-globin gene domain is transcribed into a 17-kilobase polycistronic RNA. Proc Natl Acad Sci USA.

[30] Razin SV, Rynditch A, Borunova V, Ioudinkova E, Smalko V, Scherrer K (2004). The 33 kb transcript of the chicken alpha-globin gene domain is part of the nuclear matrix. J Cell Biochem.

[31] Arriaga-Canon C, Fonseca-Guzman Y, Valdes-Quezada C, Arzate-Mejia R, Guerrero G, Recillas-Targa F (2014). A long non-coding RNA promotes full activation of adult gene expression in the chicken alpha-globin domain. Epigenetics.

[32] Razin SV, Vassetzky JYS, Kvartskhava AI, Grinenko NF, Georgiev GP (1991). Transcriptional enhancer in the vicinity of replication origin within the 5′ region of the chicken alpha-globin gene domain. J Mol Biol.

[33] Razin SV, De Moura Gallo CV, Scherrer K (1994). Characterization of the chromatin structure in the upstream area of the chicken alpha-globin gene domain. Mol Gen Genet.

[34] Knezetic J, Felsenfeld G (1989). Identification and characterization of a chicken alpha-globin enhancer. Mol Cell Biol.

[35] Higgs DR, Vernimmen D, De Gobbi M, Anguita E, Hughes J, Buckle V, et al. How transcriptional and epigenetic programmes are played out on an individual mammalian gene cluster during lineage commitment and differentiation. Biochemical Society Symposia. 2006. pp. 11–22 10.1042/bss0730011 16626283

[36] Dostie J, Dekker J (2007). Mapping networks of physical interactions between genomic elements using 5C technology. Nat Protoc.

[37] Lieberman-Aiden E, van Berkum NL, Williams L, Imakaev M, Ragoczy T, Telling A (2009). Comprehensive mapping of long-range interactions reveals folding principles of the human genome. Science.

[38] Imakaev M, Fudenberg G, McCord RP, Naumova N, Goloborodko A, Lajoie BR (2012). Iterative correction of Hi-C data reveals hallmarks of chromosome organization. Nat Methods.

[39] Gushchanskaya ES, Artemov AV, Ulyanov SV, Logacheva MD, Penin AA, Kotova ES (2014). The clustering of CpG islands may constitute an important determinant of the 3D organization of interphase chromosomes. Epigenetics.

[40] Alber F, Dokudovskaya S, Veenhoff LM, Zhang W, Kipper J, Devos D (2007). Determining the architectures of macromolecular assemblies. Nature.

[41] Filippova D, Patro R, Duggal G, Kingsford C (2014). Identification of alternative topological domains in chromatin. Algorithms Mol Biol.

[42] Sanborn AL, Rao SS, Huang SC, Durand NC, Huntley MH, Jewett A (2015). Chromatin extrusion explains key features of loop and domain formation in wild-type and engineered genomes. PNAS.

[43] Dryden NH, Broome LR, Dudbridge F, Johnson N, Orr N, Schoenfelder S (2014). Unbiased analysis of potential targets of breast cancer susceptibility loci by Capture Hi-C. Genome Res.

[44] Flint J, Tufarelli C, Peden J, Clark K, Daniels RJ, Hardison R (2001). Comparative genome analysis delimits a chromosomal domain and identifies key regulatory elements in the alpha globin cluster. Hum Mol Genet.

[45] Vyas P, Vickers MA, Simmons DL, Ayyub H, Craddock CF, Higgs DR (1992). Cis-acting sequences regulating expression of the human alpha-globin cluster lie within constitutively open chromatin. Cell.

[46] Addya S, Keller MA, Delgrosso K, Ponte CM, Vadigepalli R, Gonye GE (2004). Erythroid-induced commitment of K562 cells results in clusters of differentially expressed genes enriched for specific transcription regulatory elements. Physiol Genomics.

[47] Kowalczyk MS, Hughes JR, Babbs C, Sanchez-Pulido L, Szumska D, Sharpe JA (2012). Nprl3 is required for normal development of the cardiovascular system. Mamm Genome.

[48] Dutchak PA, Laxman S, Estill SJ, Wang C, Wang Y, Wang Y (2015). Regulation of hematopoiesis and methionine homeostasis by mTORC1 inhibitor NPRL2. Cell Rep.

[49] Kowalczyk MS, Hughes JR, Garrick D, Lynch MD, Sharpe JA, Sloane-Stanley JA (2012). Intragenic enhancers act as alternative promoters. Mol Cell.

[50] Hardison RC (2012). Evolution of hemoglobin and its genes. Cold Spring Harb Perspect Med.

[51] Travers A (1999). Chromatin modification by DNA tracking. Proc Natl Acad Sci USA.

[52] Ea V, Baudement MO, Lesne A, Forne T (2015). Contribution of topological domains and loop formation to 3D chromatin organization. Genes (Basel).

[53] de Wit E, Vos ES, Holwerda SJ, Valdes-Quezada C, Verstegen MJ, Teunissen H (2015). CTCF binding polarity determines chromatin looping. Mol Cell.

[54] Vietri Rudan M, Barrington C, Henderson S, Ernst C, Odom DT, Tanay A (2015). Comparative Hi-C reveals that CTCF underlies evolution of chromosomal domain architecture. Cell Rep.

[55] Kubo N, Ishii H, Gorkin D, Meitinger F, Xiong X, Fang R (2016). Preservation of chromatin organization after acute loss of CTCF in mouse embryonic stem cells. bioRxiv.

[56] Ulianov SV, Khrameeva EE, Gavrilov AA, Flyamer IM, Kos P, Mikhaleva EA (2016). Active chromatin and transcription play a key role in chromosome partitioning into topologically associating domains. Genome Res.

[57] Dixon JR, Jung I, Selvaraj S, Shen Y, Antosiewicz-Bourget JE, Lee AY (2015). Chromatin architecture reorganization during stem cell differentiation. Nature.

[58] Hagege H, Klous P, Braem C, Splinter E, Dekker J, Cathala G (2007). Quantitative analysis of chromosome conformation capture assays (3C-qPCR). Nat Protoc.

[59] Braem C, Recolin B, Rancourt RC, Angiolini C, Barthes P, Branchu P (2008). Genomic matrix attachment region and chromosome conformation capture quantitative real time PCR assays identify novel putative regulatory elements at the imprinted Dlk1/Gtl2 locus. J Biol Chem.

[60] Lajoie BR, van Berkum NL, Sanyal A, Dekker J (2009). My5C: web tools for chromosome conformation capture studies. Nat Methods.

[61] Langmead B, Salzberg SL (2012). Fast gapped-read alignment with Bowtie 2. Nat Methods.

[62] Cock PJ, Antao T, Chang JT, Chapman BA, Cox CJ, Dalke A (2009). Biopython: freely available Python tools for computational molecular biology and bioinformatics. Bioinformatics.

[63] Sandelin A, Alkema W, Engstrom P, Wasserman WW, Lenhard B (2004). JASPAR: an open-access database for eukaryotic transcription factor binding profiles. Nucleic Acids Res.

[64] Pettersen EF, Goddard TD, Huang CC, Couch GS, Greenblatt DM, Meng EC (2004). UCSF Chimera–a visualization system for exploratory research and analysis. J Comput Chem.

[65] Kim D, Pertea G, Trapnell C, Pimentel H, Kelley R, Salzberg SL (2013). TopHat2: accurate alignment of transcriptomes in the presence of insertions, deletions and gene fusions. Genome Biol.

[66] Anders S, Pyl PT, Huber W (2015). HTSeq—a Python framework to work with high-throughput sequencing data. Bioinformatics.

[67] Quinlan AR, Hall IM (2010). BEDTools: a flexible suite of utilities for comparing genomic features. Bioinformatics.

