# Supplementary figures and images for: Activation of the alpha-globin gene expression correlates with dramatic upregulation of nearby non-globin genes and changes in local and large-scale chromatin spatial structure
Source: Epigenetics Chromatin. 2017 Jul 11;10:35. doi: 10.1186/s13072-017-0142-4 (PMC5504709; doi:10.1186/s13072-017-0142-4)

Supplemental Figure S1

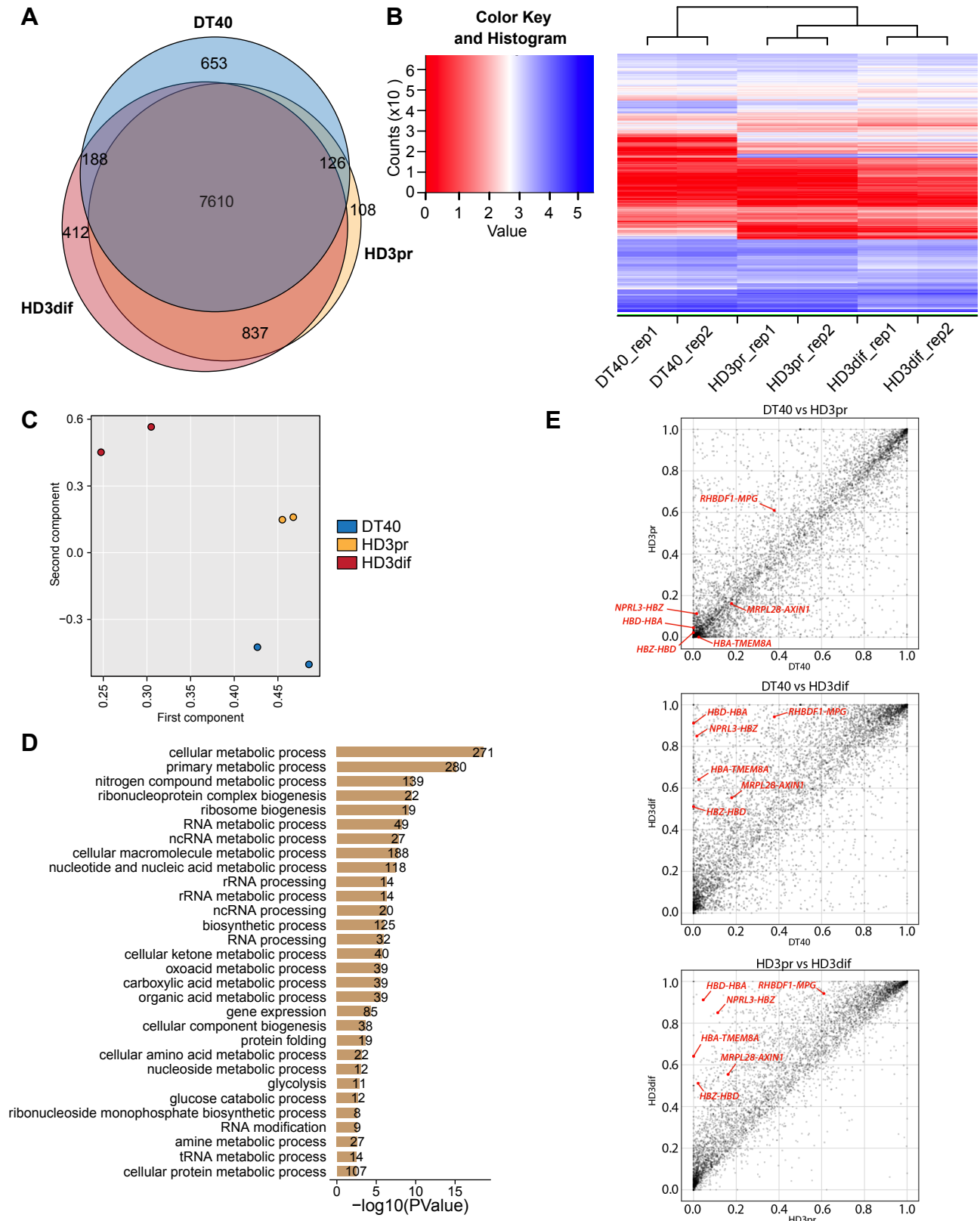

Supplement: Supplementary file 1 — Additional file 1: Figure S1. Analysis of the total rRNA-depleted RNA-seq data in the three studied cell types. (A) The Venn diagram showing the numbers of active genes shared between the studied cell types. (B) Cluster analysis of biological replicates based on total rRNA-depleted RNA-seq data. (C) Principal component analysis of the RNA-seq data. (D) Top-30 gene ontology terms for the genes downregulated (logFC < −0.6, FDR < 10−7) in differentiated HD3 cells compared to proliferating HD3 cells. The number of genes in each term is shown. (E) Scatter plots showing normalized level of transcription within intergenic regions genome-wide in the studied cell types. [file 13072_2017_142_MOESM1_ESM.pdf]

Supplemental Figure S2.

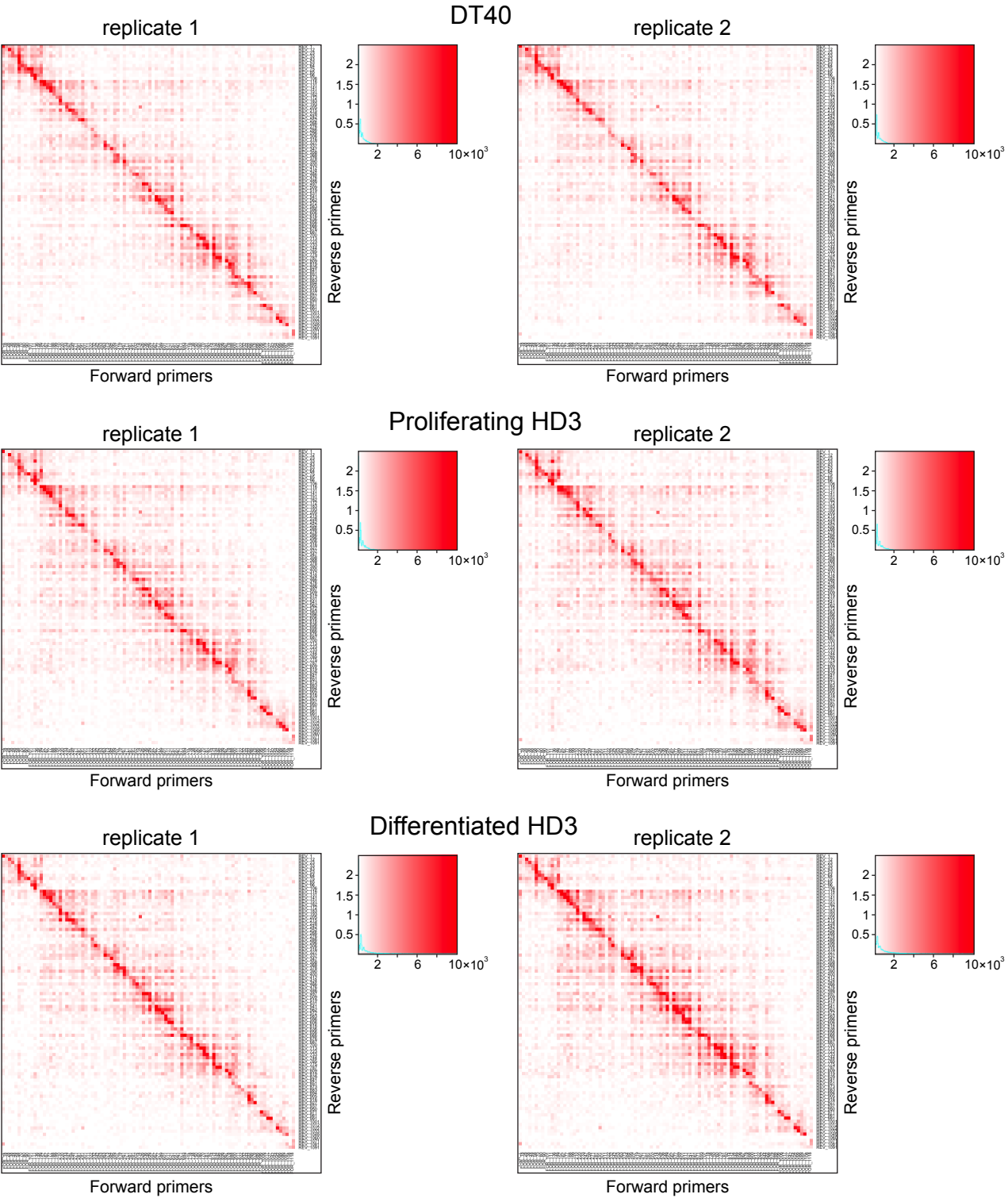

Supplement: Supplementary file 3 — Additional file 3: Figure S2. Biological replicates of the 5C experiment. Color intensity in each cell of the heatmaps represents the interaction frequency of two corresponding forward and reverse 5C primers. Histograms of the interaction counts are shown to the right of the heatmaps. [file 13072_2017_142_MOESM3_ESM.pdf]

Supplemental Figure S3.

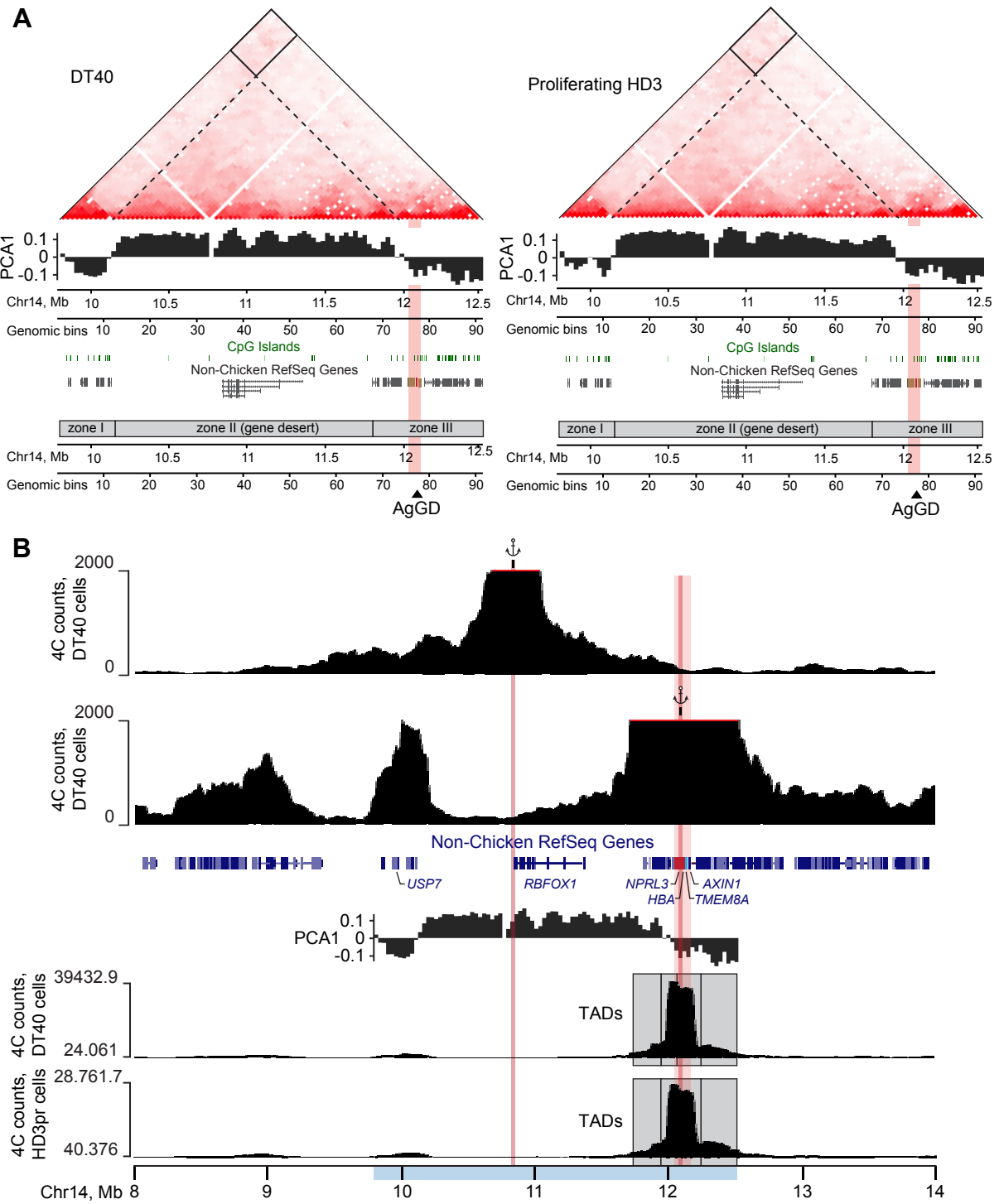

Supplement: Supplementary file 5 — Additional file 5: Figure S3. The chromatin compartment profile along the studied genomic region. (A) Heatmaps demonstrating an increased interaction frequency between gene-rich zones I and III in both lymphoid and erythroid cells. A-like and B-like chromatin compartments are outlined using black opened rectangle and black dotted triangle, respectively. The first principal component is shown below the heatmaps. (B) The 4C profiles from [39] revealing an increased interaction frequency of the alpha-globin gene domain (zone III of the studied region) with the USP7-ABAT locus (zone I), and the spatial separation of the gene desert from flanking gene-dense areas in proliferating HD3 and DT40 cells. Positions of the anchor primers are shown using red vertical lines; the alpha-globin gene domain is highlighted with a vertical pink rectangle. The first principal component (PCA1) of the 5C data (DT40 cells) is shown below the 4C profiles. The blue rectangle below the genomic coordinates track represents the genomic region analyzed in this work. The scale is changed at the bottom of the panel to emphasize the borders of 4C counts peak around the anchor. Positions of TADs that were identified using the 5C analysis are highlighted using gray rectangles. [file 13072_2017_142_MOESM5_ESM.pdf]

Supplemental Figure S4.

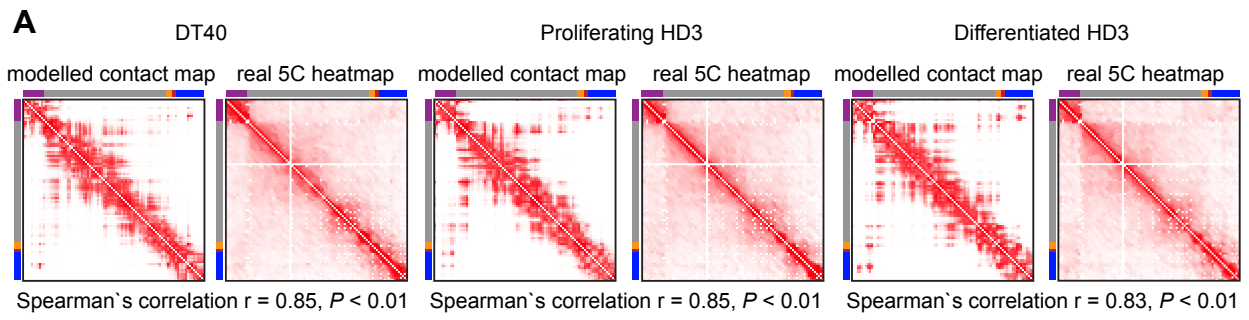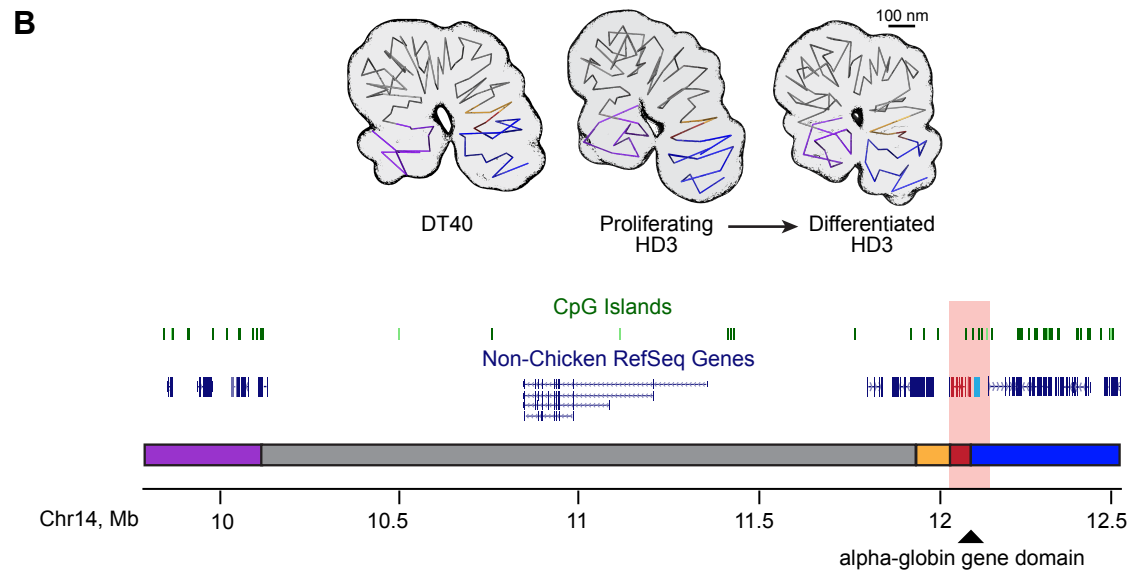

Supplement: Supplementary file 6 — Additional file 6: Figure S4. (A) Interaction heatmaps of the simulated polymer calculated with TADbit. Color bars in the heatmaps represent arbitrary partitioning of the studied region into several distinct fragments. (B) IMP-derived 3D models of the studied genome region. The central wireframe colored as the color bar in the map of the studied region represents the centroid model for simulations. The surface represents the Gaussian approximation of 1000 simulated models. [file 13072_2017_142_MOESM6_ESM.pdf]
