# Supplementary material for: Activation of the alpha-globin gene expression correlates with dramatic upregulation of nearby non-globin genes and changes in local and large-scale chromatin spatial structure
Source: Epigenetics Chromatin. 2017 Jul 11;10:35. doi: 10.1186/s13072-017-0142-4 (PMC5504709; doi:10.1186/s13072-017-0142-4)
Supplement: Supplementary file 4 — Additional file 4: Table S2. Sequences and characteristics of the 5C primers used in this study. 5C primers were designed using the alternating scheme in my5C.primers and chicken reference genome assembly galGal4. [file 13072_2017_142_MOESM4_ESM.pdf]

Table S2. Sequences and characteristics of the 5C primers used in this study.

PRIMER_NAME	P_STARTPOS	P_ENDPOS	P_SPECIFIC	P_SPECIFIC_SIZE	P_TM	P_GC	F_SIZE	P_MER	PRIMER_SEQUENCE
FOR_7	9817484	9817513	TCAATATATTTTTCTATTCCTATTCTGAAG	30	51.9	23.33	1331	89	TAATACGACTCACTATAGCCTCAATATATTTTTCTATTCCTATTCTGAAG
FOR_18	9850374	9850403	CACAAAGTGTTAGAAGTCACAGAGCATAAG	30	59.52	40	2944	41	TAATACGACTCACTATAGCCCACAAAGTGTTAGAAGTCACAGAGCATAAG
FOR_28	9884737	9884766	GAAGTTGTAAGAGAGATGGCCCTTACAAAG	30	60.82	43.33	10059	29	TAATACGACTCACTATAGCCGAAGTTGTAAGAGAGATGGCCCTTACAAAG
FOR_38	9922914	9922943	TGTTTCTTTGCCTGTGCAGCAGATGAGAAG	30	64.85	46.67	4107	118	TAATACGACTCACTATAGCCTGTTTCTTTGCCTGTGCAGCAGATGAGAAG
FOR_48	9946272	9946301	CTCTTGTGTGTACAAGGTTGTTCCATTAAG	30	59.76	40	879	36	TAATACGACTCACTATAGCCCTCTTGTGTGTACAAGGTTGTTCCATTAAG
FOR_58	9963979	9964008	CTTATATCCATTAAAGGGATCTTTTGTAAG	30	54.23	30	1104	41	TAATACGACTCACTATAGCCCTTATATCCATTAAAGGGATCTTTTGTAAG
FOR_70	9980651	9980680	TGTGAGGAATCTGCAGTGTATACAAAAAAG	30	59.47	36.67	2764	53	TAATACGACTCACTATAGCCTGTGAGGAATCTGCAGTGTATACAAAAAAG
FOR_80	10024781	10024810	CAGGGATGGGAACTGTTCAGATTTCAGAAG	30	62.59	46.67	10797	92	TAATACGACTCACTATAGCCCAGGGATGGGAACTGTTCAGATTTCAGAAG
FOR_90	10045513	10045542	CAGTCAGCATTCAAAACTCCCATGGGAAAG	30	63.53	46.67	2604	45	TAATACGACTCACTATAGCCCAGTCAGCATTCAAAACTCCCATGGGAAAG
FOR_101	10070133	10070162	CCAACCAGTGTTCGCTTCAACAGACTCAAG	30	64.8	50	553	18	TAATACGACTCACTATAGCCCCAACCAGTGTTCGCTTCAACAGACTCAAG
FOR_111	10078087	10078116	CTTGTTTGCTTTTCCTCTGCTGATCTAAAG	30	60.21	40	198	186	TAATACGACTCACTATAGCCCTTGTTTGCTTTTCCTCTGCTGATCTAAAG
FOR_136	10173833	10173862	TGATTCAGTAGGAAAAAAATCTTTCAGAAG	30	56.3	30	12274	209	TAATACGACTCACTATAGCCTGATTCAGTAGGAAAAAAATCTTTCAGAAG
FOR_146	10194636	10194665	TTTCAAATGAGAATGGCCTATTGCTGCAAG	30	62.11	40	4505	31	TAATACGACTCACTATAGCCTTTCAAATGAGAATGGCCTATTGCTGCAAG
FOR_157	10219603	10219632	TATTAGTCAGCAATAGATGTAAGAGAGAAG	30	55.93	33.33	4576	60	TAATACGACTCACTATAGCCTATTAGTCAGCAATAGATGTAAGAGAGAAG
FOR_167	10239311	10239340	AGCAAGGTGGTTTAAGCATCCACAAGGAAG	30	64.69	46.67	1427	37	TAATACGACTCACTATAGCCAGCAAGGTGGTTTAAGCATCCACAAGGAAG
FOR_177	10270378	10270407	CTACTTTGTTCAGCAATGTGTTCAGAAAAG	30	58.98	36.67	778	62	TAATACGACTCACTATAGCCCTACTTTGTTCAGCAATGTGTTCAGAAAAG
FOR_188	10295701	10295730	AAAAATGCTGGAGAGAACATGAGGTGCAAG	30	63.32	43.33	881	60	TAATACGACTCACTATAGCCAAAAATGCTGGAGAGAACATGAGGTGCAAG
FOR_199	10313087	10313116	GTGGCTATAAATCTTCCCCCCGAACCAAG	29	64.32	51.72	1567	19	TAATACGACTCACTATAGCCTGTGGCTATAAATCTTCCCCCCGAACCAAG
FOR_210	10327982	10328011	AATTTTTTTCCAGAACTGCCAAATGAAAAG	30	58.28	30	865	100	TAATACGACTCACTATAGCCAATTTTTTTCCAGAACTGCCAAATGAAAAG
FOR_220	10344618	10344647	GACTGATGTTTTGCTTGAGATAATACAAAG	30	56.89	33.33	190	51	TAATACGACTCACTATAGCCGACTGATGTTTTGCTTGAGATAATACAAAG
FOR_233	10363631	10363660	CATAGCATCATACAGTAACTTCAAGAAAAG	30	56.33	33.33	3169	57	TAATACGACTCACTATAGCCCATAGCATCATACAGTAACTTCAAGAAAAG
FOR_247	10389003	10389032	CCCTCAGTATATACACTCAGTAAACACAAG	30	58.33	40	521	41	TAATACGACTCACTATAGCCCCCTCAGTATATACACTCAGTAAACACAAG
FOR_259	10412473	10412502	CTCAGCTGCAAATTAAAGTGACTGCTCAAG	30	62	43.33	330	70	TAATACGACTCACTATAGCCCTCAGCTGCAAATTAAAGTGACTGCTCAAG
FOR_271	10433896	10433925	CAGGAAAAACAAACTTTTGGGAAAAAAAAG	30	57.42	30	2934	271	TAATACGACTCACTATAGCCCAGGAAAAACAAACTTTTGGGAAAAAAAAG
FOR_281	10447934	10447963	GGCCGCACAGATTTTGGAGAGCTAAG	26	63.47	53.85	360	41	TAATACGACTCACTATAGCCCTTCGGCCGCACAGATTTTGGAGAGCTAAG
FOR_291	10466192	10466221	AACCATACTAAACACAGCAGCTGCAAGAAG	30	63.01	43.33	588	142	TAATACGACTCACTATAGCCAACCATACTAAACACAGCAGCTGCAAGAAG
FOR_301	10483841	10483870	CTTGTTCTTAACTCTCATTCTGAAGACAAG	30	57.67	36.67	140	68	TAATACGACTCACTATAGCCCTTGTTCTTAACTCTCATTCTGAAGACAAG
FOR_311	10496700	10496729	TGGACTAAATTAAACCTTAAAAGATCAAAG	30	54.63	26.67	143	66	TAATACGACTCACTATAGCCTGGACTAAATTAAACCTTAAAAGATCAAAG
FOR_322	10519142	10519171	ATGAACGAGGAAGACAGATGTTGTCTTAAG	30	60.33	40	1320	42	TAATACGACTCACTATAGCCATGAACGAGGAAGACAGATGTTGTCTTAAG
FOR_332	10550525	10550554	CAACAAAATGCTCAATTCATGTGTGAAAAG	30	58.56	33.33	717	58	TAATACGACTCACTATAGCCCAACAAAATGCTCAATTCATGTGTGAAAAG
FOR_342	10568355	10568384	GCACAGTGGGCCATATCCTTTTCCATTAAG	30	63.31	46.67	1175	49	TAATACGACTCACTATAGCCGCACAGTGGGCCATATCCTTTTCCATTAAG
FOR_353	10587128	10587157	CTAAAAAACACCTGAAATACTTTTTAAAAG	30	52.92	23.33	1241	162	TAATACGACTCACTATAGCCCTAAAAAACACCTGAAATACTTTTTAAAAG
FOR_363	10607694	10607723	CTAGCAAGGTCTCAAAATGAAACCACAAAG	30	60.39	40	1455	75	TAATACGACTCACTATAGCCCTAGCAAGGTCTCAAAATGAAACCACAAAG
FOR_373	10622279	10622308	CACTGTGACCACACAGGCTTCCAAG	25	63.42	56	1685	54	TAATACGACTCACTATAGCCTGAAGCACTGTGACCACACAGGCTTCCAAG
FOR_384	10652355	10652384	CTGTTAACCCGGTCCTTCAGTAAATGCAAG	30	63.01	46.67	2028	51	TAATACGACTCACTATAGCCCTGTTAACCCGGTCCTTCAGTAAATGCAAG
FOR_395	10674973	10675002	GTTCGCATCAAAATAAGAATAACTTCCAAG	30	57.29	33.33	1202	64	TAATACGACTCACTATAGCCGTTCGCATCAAAATAAGAATAACTTCCAAG
FOR_407	10706927	10706956	ATCTGTAAGCCTTAGGAATATAAACACAAG	30	56.98	33.33	8167	39	TAATACGACTCACTATAGCCATCTGTAAGCCTTAGGAATATAAACACAAG
FOR_419	10729186	10729215	TTTTTTTTTAACTATAATTCTGTTATAAAG	30	48.6	13.33	1125	172	TAATACGACTCACTATAGCCTTTTTTTTTAACTATAATTCTGTTATAAAG
FOR_461	10805718	10805747	CATCTTCCTGAACAACAATTGAGTCTGAAG	30	59.8	40	2895	46	TAATACGACTCACTATAGCCCATCTTCCTGAACAACAATTGAGTCTGAAG
FOR_471	10836746	10836775	AAACTGCTTATTTCTGCCTTTGCACTGAAG	30	62.12	40	336	139	TAATACGACTCACTATAGCCAAACTGCTTATTTCTGCCTTTGCACTGAAG
FOR_481	10863272	10863301	ACCATCACCTTGATTGCACTGTACAGTAAG	30	62.31	43.33	3087	47	TAATACGACTCACTATAGCCACCATCACCTTGATTGCACTGTACAGTAAG
FOR_491	10884121	10884150	ACATAATTTTACAGCTTTTGTATTCTGAAG	30	55.04	26.67	317	105	TAATACGACTCACTATAGCCACATAATTTTACAGCTTTTGTATTCTGAAG
FOR_502	10905477	10905506	TTGGAAAACAAAGACTGAACATTGATAAAG	30	57.03	30	105	81	TAATACGACTCACTATAGCCTTGGAAAACAAAGACTGAACATTGATAAAG
FOR_514	10934274	10934303	TACAGATAGATATTTTTGTTGTTGTCTAAG	30	53.83	26.67	2304	251	TAATACGACTCACTATAGCCTACAGATAGATATTTTTGTTGTTGTCTAAG
FOR_526	10962129	10962158	GATAAATTATATGGAACAGGAGTGGAGAAG	30	57.03	36.67	561	48	TAATACGACTCACTATAGCCGATAAATTATATGGAACAGGAGTGGAGAAG
FOR_536	11001537	11001566	TGCAAGGTAGGTCTTAGAGACAGAATTAAG	30	59.67	40	4208	37	TAATACGACTCACTATAGCCTGCAAGGTAGGTCTTAGAGACAGAATTAAG
FOR_546	11026521	11026550	GAATCCCAATAAATCTTATCATAAGAGAAG	30	54.06	30	540	43	TAATACGACTCACTATAGCCGAATCCCAATAAATCTTATCATAAGAGAAG
FOR_557	11055968	11055997	TTGGAATACAGAACAGGTCTGTCACATAAG	30	60.22	40	6598	50	TAATACGACTCACTATAGCCTTGGAATACAGAACAGGTCTGTCACATAAG
FOR_567	11084996	11085025	TTGACTGAAGTCTTTGACAATAAATATAAG	30	54.22	26.67	3057	64	TAATACGACTCACTATAGCCTTGACTGAAGTCTTTGACAATAAATATAAG
FOR_577	11108891	11108920	TTATTTTCAACTTCATTATCAACAGTTAAG	30	53.3	23.33	488	76	TAATACGACTCACTATAGCCTTATTTTCAACTTCATTATCAACAGTTAAG
FOR_589	11127384	11127413	TGAAAACAAAAAGTCTGAAGCTACTTAAAG	30	56.73	30	416	66	TAATACGACTCACTATAGCCTGAAAACAAAAAGTCTGAAGCTACTTAAAG
FOR_601	11148093	11148122	TGCTATTAAATGACCAGACCTCTAAACAAG	30	58.79	36.67	752	22	TAATACGACTCACTATAGCCTGCTATTAAATGACCAGACCTCTAAACAAG
FOR_611	11172876	11172905	GTTGCAGGATGGGGAAGAAGTGAACACAAG	30	64.91	50	1966	81	TAATACGACTCACTATAGCCGTTGCAGGATGGGGAAGAAGTGAACACAAG
FOR_621	11207530	11207559	GCAAGCTGCCACCTCCACTCAAG	23	63.94	60.87	188	50	TAATACGACTCACTATAGCCTCCTGTTGCAAGCTGCCACCTCCACTCAAG
FOR_641	11253380	11253409	TTTGCTGCCGTTGTCGTATATTACTACAAG	30	60.85	40	1807	17	TAATACGACTCACTATAGCCTTTGCTGCCGTTGTCGTATATTACTACAAG
FOR_661	11291249	11291278	CAGTTTTAAGTATATATTCCTTAAAAGAAG	30	51.1	23.33	248	61	TAATACGACTCACTATAGCCCAGTTTTAAGTATATATTCCTTAAAAGAAG
FOR_671	11332567	11332596	CAAACTTTTTCAGAACTACAGCTATGAAAG	30	56.98	33.33	5662	90	TAATACGACTCACTATAGCCCAAACTTTTTCAGAACTACAGCTATGAAAG
FOR_693	11369140	11369169	ATGAAAAACTTCAAAGTGAAATAATCTAAG	30	53.51	23.33	1026	99	TAATACGACTCACTATAGCCATGAAAAACTTCAAAGTGAAATAATCTAAG
FOR_707	11383842	11383871	TTTTATAATACTTTATTCTTAATTTCAAAG	30	48.25	13.33	1151	94	TAATACGACTCACTATAGCCTTTTATAATACTTTATTCTTAATTTCAAAG
FOR_718	11405779	11405808	TTCAATGAAAGACACAATTTAACTGAAAAG	30	55.69	26.67	886	60	TAATACGACTCACTATAGCCTTCAATGAAAGACACAATTTAACTGAAAAG
FOR_738	11440296	11440325	AGCCTAATGAAGCATATTCAGATAGTGAAG	30	58.55	36.67	9171	47	TAATACGACTCACTATAGCCAGCCTAATGAAGCATATTCAGATAGTGAAG
FOR_750	11472465	11472494	GCAAATAGACTGTAGCCTTTAGAAAGTAAG	30	57.6	36.67	981	33	TAATACGACTCACTATAGCCGCAAATAGACTGTAGCCTTTAGAAAGTAAG
FOR_760	11491139	11491168	ACTACTACTTAACAAATAACCGTTTTCAAG	30	55.99	30	3250	33	TAATACGACTCACTATAGCCACTACTACTTAACAAATAACCGTTTTCAAG
FOR_770	11515180	11515209	CTTCAGATATGAATGGTATTTTTTCAAAAG	30	54	26.67	2627	66	TAATACGACTCACTATAGCCCTTCAGATATGAATGGTATTTTTTCAAAAG
FOR_781	11536061	11536090	CACAGCAGCACAGCCCCAAAG	21	62.61	61.9	2428	413	TAATACGACTCACTATAGCCGACAGTCCCCACAGCAGCACAGCCCCAAAG
FOR_792	11568640	11568669	AGATGACTGAGTCAAAAAGGAATTAGGAAG	30	58.78	36.67	5295	58	TAATACGACTCACTATAGCCAGATGACTGAGTCAAAAAGGAATTAGGAAG
FOR_814	11623537	11623566	ATTGTCAAGATGAAACACTGTCCGAAAAAG	30	60.14	36.67	4355	42	TAATACGACTCACTATAGCCATTGTCAAGATGAAACACTGTCCGAAAAAG
FOR_834	11666949	11666978	AACACAGGAATCTTCAGATATGGTACAAAG	30	58.81	36.67	2895	41	TAATACGACTCACTATAGCCAACACAGGAATCTTCAGATATGGTACAAAG
FOR_846	11701673	11701702	AATAAAGAAATAAATAAATTTAAAGAGAAG	30	48.46	13.33	9218	436	TAATACGACTCACTATAGCCAATAAAGAAATAAATAAATTTAAAGAGAAG
FOR_856	11724321	11724350	CTGGCAAAACAATGACAGCACAGAAGAAAG	30	62.55	43.33	1299	115	TAATACGACTCACTATAGCCCTGGCAAAACAATGACAGCACAGAAGAAAG
FOR_867	11741888	11741917	CAAGAGCCCAAACACTCACAACTGAAAAAG	30	62.33	43.33	680	37	TAATACGACTCACTATAGCCCAAGAGCCCAAACACTCACAACTGAAAAAG
FOR_878	11771739	11771768	GATCAAATCATCATTCAGATAAATGAAAAG	30	53.89	26.67	136	80	TAATACGACTCACTATAGCCGATCAAATCATCATTCAGATAAATGAAAAG
FOR_888	11813017	11813046	TGTGCTTGAATTCCTTGCAGATCTTGCAAG	30	63.63	43.33	9191	62	TAATACGACTCACTATAGCCTGTGCTTGAATTCCTTGCAGATCTTGCAAG
FOR_900	11838305	11838334	TGAATTATGAGGAACCTTGCAAGTATAAAG	30	57.54	33.33	116	29	TAATACGACTCACTATAGCCTGAATTATGAGGAACCTTGCAAGTATAAAG
FOR_911	11857460	11857489	TACCATTGACATGAGGAGGATGAGGAGAAG	30	62.7	46.67	6709	157	TAATACGACTCACTATAGCCTACCATTGACATGAGGAGGATGAGGAGAAG
FOR_922	11882060	11882089	CTTAAAAGGCCAGTGGGGATGCAACAAG	28	63.91	50	1809	33	TAATACGACTCACTATAGCCACCTTAAAAGGCCAGTGGGGATGCAACAAG
FOR_932	11903510	11903539	ATAGCATACCTCCAGACAAGGTTCCTGAAG	30	63.06	46.67	350	42	TAATACGACTCACTATAGCCATAGCATACCTCCAGACAAGGTTCCTGAAG
FOR_944	11922861	11922890	AGCATCTTTCAGCATCCTGACATTCAGAAG	30	62.59	43.33	1350	74	TAATACGACTCACTATAGCCAGCATCTTTCAGCATCCTGACATTCAGAAG
FOR_966	11959742	11959771	TTTATGAGGTTTGATGTGATTAATCAAAAG	30	55.08	26.67	568	34	TAATACGACTCACTATAGCCTTTATGAGGTTTGATGTGATTAATCAAAAG
FOR_976	12000724	12000753	CACAGAGGAGGGAGCAGCAGGAAG	24	64.41	62.5	301	139	TAATACGACTCACTATAGCCTTGGTTCACAGAGGAGGGAGCAGCAGGAAG
FOR_986	12028668	12028697	GTGAGGCAAAGCCAAAAACCCACAGAAAG	29	64.43	48.28	1130	126	TAATACGACTCACTATAGCCCGTGAGGCAAAGCCAAAAACCCACAGAAAG
FOR_996	12060214	12060243	TGAAATATTCAGTGATTTAACTGAATCAAG	30	54.66	26.67	1696	70	TAATACGACTCACTATAGCCTGAAATATTCAGTGATTTAACTGAATCAAG
FOR_1006	12083275	12083304	TTGTGCTTAGGGAACTCAGCTCCCTAAAAG	30	63.71	46.67	539	42	TAATACGACTCACTATAGCCTTGTGCTTAGGGAACTCAGCTCCCTAAAAG
FOR_1017	12128317	12128346	GCTCTGCTGGTGTTGGCAGCAAG	23	64.58	60.87	6577	80	TAATACGACTCACTATAGCCAACGGGAGCTCTGCTGGTGTTGGCAGCAAG
FOR_1027	12158967	12158996	AACGCTCTCCCCAAGTGGCAGAAG	24	64.84	58.33	5759	36	TAATACGACTCACTATAGCCCCACGCAACGCTCTCCCCAAGTGGCAGAAG

FOR_1042	12203436	12203465	GGTCTCCTCTAAGGAAAACGGCAATAAAAG	30	61.2	43.33	5724	32	TAATACGACTCACTATAGCCGGTCTCCTCTAAGGAAAACGGCAATAAAAG
FOR_1054	12224460	12224489	AGAACTAAAAGCACTTTGACCATTAAAAAG	30	57.04	30	1219	41	TAATACGACTCACTATAGCCAGAACTAAAAGCACTTTGACCATTAAAAAG
FOR_1066	12264782	12264811	GGGTTTCATACTGGGTCCGATTTTAGAAAG	30	61.08	43.33	455	19	TAATACGACTCACTATAGCCGGGTTTCATACTGGGTCCGATTTTAGAAAG
FOR_1086	12299460	12299489	CCCTTTGTGCCTCCCTGCCAAG	22	64.07	63.64	631	48	TAATACGACTCACTATAGCCAAACGGGTCCCTTTGTGCCTCCCTGCCAAG
FOR_1097	12334437	12334466	GGCTGTGTGCAAGCCAAACCAAG	23	63.19	56.52	725	34	TAATACGACTCACTATAGCCAGAACTCGGCTGTGTGCAAGCCAAACCAAG
FOR_1108	12363546	12363575	TCCAGAGATACTACAGACACAAAAATGAAG	30	58.5	36.67	341	80	TAATACGACTCACTATAGCCTCCAGAGATACTACAGACACAAAAATGAAG
FOR_1118	12379474	12379503	AGCATCTCTGTGCCTGCTCCCAAAAG	26	64.94	53.85	720	153	TAATACGACTCACTATAGCCATACAGCATCTCTGTGCCTGCTCCCAAAAG
REV_1	9802831	9802860	CTTTACGCTGAATTTTTGCCTCTAGCAGTG	30	61.99	43.33	2860	57	CTTTACGCTGAATTTTTGCCTCTAGCAGTGTCCCTTTAGTGAGGGTTAATA
REV_12	9835286	9835315	CTTCCAAGGAGGTCTGACACTGAACTAATG	30	62.07	46.67	1637	34	CTTCCAAGGAGGTCTGACACTGAACTAATGTCCCTTTAGTGAGGGTTAATA
REV_23	9860127	9860156	CTTTGATGTAGGAATTCAGATTGGCTATCA	30	58.92	36.67	3180	41	CTTTGATGTAGGAATTCAGATTGGCTATCATCCCTTTAGTGAGGGTTAATA
REV_33	9898173	9898202	CTTCTGCTCACACACTCACCATCTCTTCTT	30	63.52	46.67	2227	63	CTTCTGCTCACACACTCACCATCTCTTCTTTCCCTTTAGTGAGGGTTAATA
REV_43	9938315	9938344	CTTTCCTAATCAGCTCTGAAAAGTAACTAC	30	57.38	36.67	289	72	CTTTCCTAATCAGCTCTGAAAAGTAACTACTCCCTTTAGTGAGGGTTAATA
REV_53	9959113	9959142	CTTATTCCAAAACTGAAAATCAAGGTTTGT	30	57.28	30	7079	109	CTTATTCCAAAACTGAAAATCAAGGTTTGTTCCCTTTAGTGAGGGTTAATA
REV_64	9973511	9973540	CTTTAGTGTAAGCTCTGCACACTGAAATAC	30	59.55	40	1818	58	CTTTAGTGTAAGCTCTGCACACTGAAATACTCCCTTTAGTGAGGGTTAATA
REV_75	9993359	9993388	CTTTGTCTCTAGGCTAAATTCAAAATGGGA	30	59.24	36.67	1117	34	CTTTGTCTCTAGGCTAAATTCAAAATGGGATCCCTTTAGTGAGGGTTAATA
REV_85	10027074	10027103	CTTAAACAAATGCTATGCCAAAGGTGGCAC	30	62.76	43.33	384	41	CTTAAACAAATGCTATGCCAAAGGTGGCACTCCCTTTAGTGAGGGTTAATA
REV_96	10061080	10061109	CTTAGCTCAGGAGTACAGATTTTCATGGTC	30	60.44	43.33	1314	64	CTTAGCTCAGGAGTACAGATTTTCATGGTCTCCCTTTAGTGAGGGTTAATA
REV_106	10075593	10075622	CTTATTTTCATCAACACAGTTTCTAGGTGT	30	57.76	33.33	2832	55	CTTATTTTCATCAACACAGTTTCTAGGTGTTCCCTTTAGTGAGGGTTAATA
REV_116	10099839	10099868	CTTGGATAATTGCAACTGTGGCCTCTCACC	30	64.83	50	702	27	CTTGGATAATTGCAACTGTGGCCTCTCACCTCCCTTTAGTGAGGGTTAATA
REV_131	10138041	10138070	CTTAGTGCTGGTCTGTGTCTGTGACTTGAC	30	63.88	50	4624	59	CTTAGTGCTGGTCTGTGTCTGTGACTTGACTCCCTTTAGTGAGGGTTAATA
REV_141	10183317	10183346	CTTTTGAGCTATATAATGTTTTGCAGCACA	30	58.64	33.33	1380	38	CTTTTGAGCTATATAATGTTTTGCAGCACATCCCTTTAGTGAGGGTTAATA
REV_151	10207291	10207320	CTTGCCATAGTTGTCAGCATTGTTGACTGA	30	63.01	43.33	986	47	CTTGCCATAGTTGTCAGCATTGTTGACTGATCCCTTTAGTGAGGGTTAATA
REV_162	10229687	10229716	CTTAAATCTCTCTTTGTACTGTTCTCGATC	30	57.13	36.67	1761	59	CTTAAATCTCTCTTTGTACTGTTCTCGATCTCCCTTTAGTGAGGGTTAATA
REV_172	10260553	10260582	CTTTCAGTGCACTGGGAAACCTGTACTGC	29	64.9	51.72	1283	72	CTTTCAGTGCACTGGGAAACCTGTACTGCTTCCCTTTAGTGAGGGTTAATA
REV_183	10287534	10287563	CTTCTATAGCTATGCATCTTCCAAATTCTG	30	57.61	36.67	367	57	CTTCTATAGCTATGCATCTTCCAAATTCTGTCCCTTTAGTGAGGGTTAATA
REV_193	10309892	10309921	CTTGTAATTTGTCACTTCAAGACATCAGTT	30	58.09	33.33	2869	44	CTTGTAATTTGTCACTTCAAGACATCAGTTTCCCTTTAGTGAGGGTTAATA
REV_205	10317906	10317935	CTTGATGTAGGAGTCAGTTTTGACACAGTG	30	60.99	43.33	129	46	CTTGATGTAGGAGTCAGTTTTGACACAGTGTCCCTTTAGTGAGGGTTAATA
REV_215	10340608	10340637	CTTTACAACATGCCATGAAGTCTCATGCTG	30	61.93	43.33	1868	38	CTTTACAACATGCCATGAAGTCTCATGCTGTCCCTTTAGTGAGGGTTAATA
REV_228	10353576	10353605	CTTAATACCTCTTCTATTCTATTTTCGTCC	30	55.23	33.33	1143	67	CTTAATACCTCTTCTATTCTATTTTCGTCCTCCCTTTAGTGAGGGTTAATA
REV_242	10378656	10378685	CTTACTGTACTTAATATGCTGAAGACAACA	30	56.98	33.33	2271	57	CTTACTGTACTTAATATGCTGAAGACAACATCCCTTTAGTGAGGGTTAATA
REV_252	10397027	10397056	CTTTGGTCCTAAATTCCACCTAGATACACT	30	59.81	40	654	33	CTTTGGTCCTAAATTCCACCTAGATACACTTCCCTTTAGTGAGGGTTAATA
REV_266	10425540	10425569	CTTCAAACTTAATGAGAAATCTCTGACTTT	30	56.07	30	312	80	CTTCAAACTTAATGAGAAATCTCTGACTTTTCCCTTTAGTGAGGGTTAATA
REV_276	10443565	10443594	CTTTGGCCACTGTGAATAGGAATCCAATTT	30	61.61	40	3621	32	CTTTGGCCACTGTGAATAGGAATCCAATTTTCCCTTTAGTGAGGGTTAATA
REV_286	10454145	10454174	CTTCTGTAAATTTCATATTTCTTTTTATTG	30	51	20	831	125	CTTCTGTAAATTTCATATTTCTTTTTATTGTCCCTTTAGTGAGGGTTAATA
REV_296	10476094	10476123	CTTGCTTGACCACACTGCCCAGC	23	64.38	60.87	277	83	CTTGCTTGACCACACTGCCCAGCACATTACTCCCTTTAGTGAGGGTTAATA
REV_306	10489388	10489417	CTTTCCATTCTGCTGAGACTCCACACACAG	30	64.23	50	1503	67	CTTTCCATTCTGCTGAGACTCCACACACAGTCCCTTTAGTGAGGGTTAATA
REV_316	10510343	10510372	CTTTCCTTGTTCAGGGAAATAACATTTACC	30	58.47	36.67	1931	76	CTTTCCTTGTTCAGGGAAATAACATTTACCTCCCTTTAGTGAGGGTTAATA
REV_327	10537323	10537352	CTTTGGAGTATGTGAAATAATACAAAAAAA	30	53.71	23.33	2164	112	CTTTGGAGTATGTGAAATAATACAAAAAAATCCCTTTAGTGAGGGTTAATA
REV_337	10558994	10559023	CTTGATGGGGCATTTAGAATGCCCTTTTCA	30	63.39	43.33	222	25	CTTGATGGGGCATTTAGAATGCCCTTTTCATCCCTTTAGTGAGGGTTAATA
REV_347	10580400	10580429	CTTCAAAGCTCTGCTCTCTGAGTTCTTTGC	30	63.05	46.67	618	115	CTTCAAAGCTCTGCTCTCTGAGTTCTTTGCTCCCTTTAGTGAGGGTTAATA
REV_358	10595037	10595066	CTTGTGTTTTGTGCCTCGTATTTGTCTGGA	30	63.08	43.33	191	26	CTTGTGTTTTGTGCCTCGTATTTGTCTGGATCCCTTTAGTGAGGGTTAATA
REV_368	10610281	10610310	CTTGTGGATTATGGATGGATCTGTTTTGGT	30	61.05	40	753	40	CTTGTGGATTATGGATGGATCTGTTTTGGTTCCCTTTAGTGAGGGTTAATA
REV_378	10641873	10641902	CTTGAAGGTTATCGATTTTTAAATGAAGAG	30	55.23	30	945	79	CTTGAAGGTTATCGATTTTTAAATGAAGAGTCCCTTTAGTGAGGGTTAATA
REV_390	10659921	10659950	CTTCTGAAAGCATGGATGGAGGGAATAGTG	30	62.5	46.67	2179	68	CTTCTGAAAGCATGGATGGAGGGAATAGTGTCCCTTTAGTGAGGGTTAATA
REV_400	10679803	10679832	CTTTGACTCATTGTTTTTAGTGCGATTCCC	30	60.57	40	882	56	CTTTGACTCATTGTTTTTAGTGCGATTCCCTCCCTTTAGTGAGGGTTAATA
REV_412	10717467	10717496	CTTCTACCCACAGAGTTTTCTCTGACTTCT	30	61.28	43.33	3227	70	CTTCTACCCACAGAGTTTTCTCTGACTTCTTCCCTTTAGTGAGGGTTAATA
REV_424	10738099	10738128	CTTGCCCACAAGACTGTGTCAACATATGCA	30	64.71	46.67	155	35	CTTGCCCACAAGACTGTGTCAACATATGCATCCCTTTAGTGAGGGTTAATA
REV_466	10828866	10828895	CTTTTCAGAGCAAAAACTGTCTTTTTGATT	30	57.75	30	235	104	CTTTTCAGAGCAAAAACTGTCTTTTTGATTTCCCTTTAGTGAGGGTTAATA
REV_476	10847191	10847220	CTTAGTCTGAAAAGGTAAAAATATGTTGGT	30	56.13	30	976	85	CTTAGTCTGAAAAGGTAAAAATATGTTGGTTCCCTTTAGTGAGGGTTAATA
REV_486	10876393	10876422	CTTTTTAAATACCTTTGTAAAGTATTTATT	30	50.13	16.67	870	93	CTTTTTAAATACCTTTGTAAAGTATTTATTTCCCTTTAGTGAGGGTTAATA
REV_497	10897186	10897215	CTTGTTATTAGTGGTTATCTCAGCTGCACT	30	60.46	40	831	37	CTTGTTATTAGTGGTTATCTCAGCTGCACTTCCCTTTAGTGAGGGTTAATA
REV_509	10926147	10926176	CTTGATACTGTAATTCAAATACCAATCATG	30	54.95	30	2180	34	CTTGATACTGTAATTCAAATACCAATCATGTCCCTTTAGTGAGGGTTAATA
REV_519	10941799	10941828	CTTGCAAAGGCATTGTGGTGCAAACGTAAG	30	64.71	46.67	1394	39	CTTGCAAAGGCATTGTGGTGCAAACGTAAGTCCCTTTAGTGAGGGTTAATA
REV_531	10989897	10989926	CTTTTCTTCAAGTGGAATTATATATAATAT	30	50.43	20	7125	70	CTTTTCTTCAAGTGGAATTATATATAATATTCCCTTTAGTGAGGGTTAATA
REV_541	11022958	11022987	CTTTCTTTTCCAGGATCTAAACAGAAATTA	30	55.96	30	114	93	CTTTCTTTTCCAGGATCTAAACAGAAATTATCCCTTTAGTGAGGGTTAATA
REV_562	11066530	11066559	CTTTTAGAGTAATTGTATTACTTCCTCTTA	30	53.04	26.67	2837	47	CTTTTAGAGTAATTGTATTACTTCCTCTTATCCCTTTAGTGAGGGTTAATA
REV_572	11097611	11097640	CTTGTCAGCGTTCAAGTGACACTTGGGTAA	30	64.29	46.67	3633	36	CTTGTCAGCGTTCAAGTGACACTTGGGTAATCCCTTTAGTGAGGGTTAATA
REV_583	11115055	11115084	CTTGTGGTGGTACTTAGTGTAAGCTGTGTG	30	62.47	46.67	1154	29	CTTGTGGTGGTACTTAGTGTAAGCTGTGTGTCCCTTTAGTGAGGGTTAATA
REV_596	11141320	11141349	CTTGAACTTTTGAATTTATTTGTTCAGAAA	30	54.4	23.33	1381	97	CTTGAACTTTTGAATTTATTTGTTCAGAAATCCCTTTAGTGAGGGTTAATA
REV_606	11159525	11159554	CTTCCAGTAATGTTAAATAAACATATTAAA	30	51.29	20	2838	102	CTTCCAGTAATGTTAAATAAACATATTAAATCCCTTTAGTGAGGGTTAATA
REV_616	11196323	11196352	CTTGTACAATATGAACTGATAACTTCTTCA	30	55.51	30	6026	51	CTTGTACAATATGAACTGATAACTTCTTCATCCCTTTAGTGAGGGTTAATA
REV_636	11244923	11244952	CTTTAAGAACTGACATCTGAAAAGTTTTGA	30	56.56	30	279	87	CTTTAAGAACTGACATCTGAAAAGTTTTGATCCCTTTAGTGAGGGTTAATA
REV_656	11283637	11283666	CTTCCCTACTTTAAACATCTTCTAAGCTAA	30	56.67	33.33	390	65	CTTCCCTACTTTAAACATCTTCTAAGCTAATCCCTTTAGTGAGGGTTAATA
REV_666	11319438	11319467	CTTTTCAAGTGACCACTCTAGAAATTCCCA	30	60.62	40	15327	31	CTTTTCAAGTGACCACTCTAGAAATTCCCATCCCTTTAGTGAGGGTTAATA
REV_676	11336808	11336837	CTTTTTAATTCATTTTTATGTCCCTTCTGT	30	55.28	26.67	467	189	CTTTTTAATTCATTTTTATGTCCCTTCTGTTCCCTTTAGTGAGGGTTAATA
REV_687	11361598	11361627	CTTATACTATAAGGAAGACATTTTAATTTT	30	50.72	20	1278	71	CTTATACTATAAGGAAGACATTTTAATTTTCCCTTTAGTGAGGGTTAATA
REV_700	11378966	11378995	CTTAAAGAGAAGAAAAAGGAAGTAACTGTT	30	56	30	1143	312	CTTAAAGAGAAGAAAAAGGAAGTAACTGTTTCCCTTTAGTGAGGGTTAATA
REV_713	11396459	11396488	CTTTTCTCACTATACCTTAGTTTTCAGTAT	30	54.94	30	1478	51	CTTTTCTCACTATACCTTAGTTTTCAGTATTCCCTTTAGTGAGGGTTAATA
REV_723	11412316	11412345	CTTTAAAAACTTAACATCTGTGGCCTTACA	30	58.16	33.33	2180	49	CTTTAAAAACTTAACATCTGTGGCCTTACATCCCTTTAGTGAGGGTTAATA
REV_733	11426484	11426513	CTTATTGGAAAGAGAATTCACTGCTGAGAA	30	59.13	36.67	971	74	CTTATTGGAAAGAGAATTCACTGCTGAGAATCCCTTTAGTGAGGGTTAATA
REV_744	11460140	11460169	CTTGCAAGTTAATTGCTGGATTTCATAGAT	30	58.37	33.33	2576	53	CTTGCAAGTTAATTGCTGGATTTCATAGATTCCCTTTAGTGAGGGTTAATA
REV_765	11502299	11502328	CTTCAGCATTTTTTTCATTATTCTGGCCAA	30	59.29	33.33	683	198	CTTCAGCATTTTTTTCATTATTCTGGCCAATCCCTTTAGTGAGGGTTAATA
REV_775	11520571	11520600	CTTGCAGGTGTAAGCAGTGCACAGTC	26	63.68	53.85	455	65	CTTGCAGGTGTAAGCAGTGCACAGTCCAAATCCCTTTAGTGAGGGTTAATA
REV_787	11549083	11549112	CTTTTCCCATAAGATAAATGAGGCTTTGGA	30	59.36	36.67	4750	68	CTTTTCCCATAAGATAAATGAGGCTTTGGATCCCTTTAGTGAGGGTTAATA
REV_809	11614269	11614298	CTTCAATTGCTGGAACACACTGAGTCATCA	30	62.79	43.33	2488	61	CTTCAATTGCTGGAACACACTGAGTCATCATCCCTTTAGTGAGGGTTAATA
REV_819	11629647	11629676	CTTGAACATCAGTATGTTCACAAATATTCA	30	56.32	30	1162	46	CTTGAACATCAGTATGTTCACAAATATTCATCCCTTTAGTGAGGGTTAATA
REV_829	11647647	11647676	CTTGAGATTCTCTTAACTATGTATTAATCA	30	53.12	26.67	1388	28	CTTGAGATTCTCTTAACTATGTATTAATCATCCCTTTAGTGAGGGTTAATA
REV_841	11682498	11682527	CTTTTGAATTATTTGCGTTGCTGCGACTGA	30	62.49	40	2707	32	CTTTTGAATTATTTGCGTTGCTGCGACTGATCCCTTTAGTGAGGGTTAATA
REV_851	11709836	11709865	CTTCCTGTGTGCTGTCAGTACAAATGGCTT	30	64.51	46.67	3056	102	CTTCCTGTGTGCTGTCAGTACAAATGGCTTTCCCTTTAGTGAGGGTTAATA
REV_873	11763847	11763876	CTTTTCAAAAGGTGCAATTTCATATTATTG	30	54.99	26.67	1723	50	CTTTTCAAAAGGTGCAATTTCATATTATTGTCCCTTTAGTGAGGGTTAATA
REV_883	11788272	11788301	CTTTTAGAAAGCTACCCAATTTATTTTAAT	30	53.43	23.33	1781	43	CTTTTAGAAAGCTACCCAATTTATTTTAATTCCCTTTAGTGAGGGTTAATA
REV_894	11830521	11830550	CTTTGCAGCCTGCCCTTTCTTTACACC	27	64.08	51.85	1723	73	CTTTGCAGCCTGCCCTTTCTTTACACCACTTCCCTTTAGTGAGGGTTAATA
REV_905	11843719	11843748	CTTATAAAGTAATCATGATTCATTCTCTGA	30	53.57	26.67	229	46	CTTATAAAGTAATCATGATTCATTCTCTGATCCCTTTAGTGAGGGTTAATA
REV_916	11867426	11867455	CTTCCAAGCTGTGAGCTGTTTGAGGACAG	29	64.69	51.72	190	91	CTTCCAAGCTGTGAGCTGTTTGAGGACAGTTCCCTTTAGTGAGGGTTAATA
REV_927	11899091	11899120	CTTGTCAGTTAATAGAACTATTTATTTCTT	30	52.34	23.33	1396	45	CTTGTCAGTTAATAGAACTATTTATTTCTTTCCCTTTAGTGAGGGTTAATA
REV_937	11910505	11910534	CTTTTCTAAACATGTTTATTTCCTTGATGT	30	55.23	26.67	1815	99	CTTTTCTAAACATGTTTATTTCCTTGATGTTCCCTTTAGTGAGGGTTAATA
REV_950	11928885	11928914	CTTAGGCTTGGTAAGTGAATTGAAATGATA	30	57.54	33.33	3890	46	CTTAGGCTTGGTAAGTGAATTGAAATGATATCCCTTTAGTGAGGGTTAATA
REV_971	11981891	11981920	CTTTTTGCCAAGGAGAAGCGGCCC	24	64.64	58.33	6084	50	CTTTTTGCCAAGGAGAAGCGGCCCATGCCGTCCCTTTAGTGAGGGTTAATA
REV_981	12024535	12024564	CTTGAAATAGCAGAACTTGAGAACGTTTGG	30	60.28	40	1104	51	CTTGAAATAGCAGAACTTGAGAACGTTTGGTCCCTTTAGTGAGGGTTAATA
REV_991	12042936	12042965	CTTTGAACTGGTCTCAAAAGCAGCTGGAAA	30	63.36	43.33	2939	91	CTTTGAACTGGTCTCAAAAGCAGCTGGAAATCCCTTTAGTGAGGGTTAATA
REV_1001	12072411	12072440	CTTGCTCAGTGCAGAGCCATTCAGAAATCT	30	64.41	46.67	1882	109	CTTGCTCAGTGCAGAGCCATTCAGAAATCTTCCCTTTAGTGAGGGTTAATA
REV_1012	12098634	12098663	CTTTCTTGTCCACCAGCACCTGTAAGAGC	29	64.55	51.72	6984	56	CTTTCTTGTCCACCAGCACCTGTAAGAGCATCCCTTTAGTGAGGGTTAATA

REV_1022	12140954	12140983	CTTTTTTGGTACTGGGGACAAATAGAAATA	30	57.72	33.33	373	57	CTTTTTTGGTACTGGGGACAAATAGAAATATCCCTTTAGTGAGGGTTAATA
REV_1033	12175334	12175363	CTTGGTATTTCAGTGAAATTACAAATTTTG	30	54.69	26.67	3555	130	CTTGGTATTTCAGTGAAATTACAAATTTTGTCCCTTTAGTGAGGGTTAATA
REV_1049	12216521	12216550	CTTTTGCAAAATCCTCCCAGCTTCTCTGCT	30	64.88	46.67	1237	98	CTTTTGCAAAATCCTCCCAGCTTCTCTGCTTCCCTTTAGTGAGGGTTAATA
REV_1060	12256623	12256652	CTTGAGCTTCTGTTTGGGGACCATTAGGC	29	64.79	51.72	2135	49	CTTGAGCTTCTGTTTGGGGACCATTAGGCTTCCCTTTAGTGAGGGTTAATA
REV_1071	12273502	12273531	CTTTTCTTCCCAAGGAAGTTCTATTTTGAT	30	57.91	33.33	1937	66	CTTTTCTTCCCAAGGAAGTTCTATTTTGATTCCCTTTAGTGAGGGTTAATA
REV_1081	12294388	12294417	CTTGTTGGGCTTGGCTGGTCCTTTTG	26	64.43	53.85	1236	31	CTTGTTGGGCTTGGCTGGTCCTTTTGGAACTCCCTTTAGTGAGGGTTAATA
REV_1091	12323402	12323431	CTTTCCTTCCTTGTAACATCAGCAACATCT	30	61.05	40	2772	68	CTTTCCTTCCTTGTAACATCAGCAACATCTTCCCTTTAGTGAGGGTTAATA
