# Supplementary material for: Activation of the alpha-globin gene expression correlates with dramatic upregulation of nearby non-globin genes and changes in local and large-scale chromatin spatial structure
Source: Epigenetics Chromatin. 2017 Jul 11;10:35. doi: 10.1186/s13072-017-0142-4 (PMC5504709; doi:10.1186/s13072-017-0142-4)
Supplement: Supplementary file 7 — Additional file 7: Table S3. Genomic coordinates of topologically associating domains within the studied region. TADs were identified using the optimal segmentation algorithm from the Lavaburst package with the with the Armatus scoring function. [file 13072_2017_142_MOESM7_ESM.pdf]

Table S3. Genomic coordinates of topologically associating domains within the studied region.

Cell type	γ value	Genomic bins recognized as boundaries															
DT40	0.08	0	11	12	50	52	65	66	72	73	76	77	82	83	91		
	0.09	0	11	12	50	52	65	66	72	73	76	77	82	83	91		
	0.1	0	11	12	50	52	65	66	72	73	76	77	82	83	91		
	0.11	0	11	12	50	52	65	66	72	73	76	77	82	83	91		
	0.12	0	11	12	50	52	65	66	72	73	76	77	82	83	91		
	0.13	0	11	12	50	52	65	66	72	73	76	77	82	83	91		
	0.14	0	11	12	50	52	65	66	72	73	76	77	82	83	91		
	0.15	0	11	12	50	52	65	66	72	73	76	77	82	83	91		
	0.16	0	11	12	50	52	65	66	72	73	76	77	82	83	91		
	0.17	0	11	12	50	52	65	66	72	73	76	77	82	83	91		
	0.18	0	11	12	50	52	65	66	72	73	76	77	82	83	91		
	0.19	0	11	12	50	52	65	66	72	73	76	77	82	83	91		
	0.2	0	11	12	50	52	65	66	72	73	76	77	82	83	91		
	0.21	0	11	12	50	52	65	66	72	73	76	77	82	83	91		
	0.22	0	11	12	50	52	65	66	72	73	76	77	82	83	91		
	0.23	0	11	12	50	52	65	66	72	73	76	77	82	83	91		
	0.24	0	11	12	50	52	65	66	72	73	76	77	82	83	91		
	0.25	0	11	12	50	52	65	66	72	73	76	77	82	83	91		
HD3pr	0.08	0	11	12	51	53	57	58	63	64	67	68	72	73	82	83	91
	0.09	0	11	12	51	53	57	58	63	64	67	68	72	73	82	83	91
	0.1	0	11	12	51	53	57	58	63	64	67	68	72	73	82	83	91
	0.11	0	11	12	51	53	57	58	63	64	67	68	72	73	82	83	91
	0.12	0	11	12	51	53	57	58	63	64	67	68	72	73	82	83	91
	0.13	0	11	12	51	53	57	58	63	64	67	68	72	73	82	83	91
	0.14	0	11	12	51	53	57	58	63	64	67	68	72	73	82	83	91
	0.15	0	11	12	51	53	57	58	63	64	67	68	72	73	82	83	91
	0.16	0	11	12	51	53	57	58	63	64	67	68	72	73	82	83	91
	0.17	0	11	12	51	53	57	58	63	64	67	68	72	73	82	83	91
	0.18	0	11	12	51	53	57	58	63	64	67	68	72	73	82	83	91
	0.19	0	11	12	51	53	57	58	63	64	67	68	72	73	82	83	91
	0.2	0	11	12	51	53	57	58	63	64	67	68	72	73	82	83	91
	0.21	0	11	12	51	53	57	58	63	64	67	68	72	73	82	83	91
	0.22	0	11	12	51	53	57	58	63	64	67	68	72	73	82	83	91
	0.23	0	11	12	51	53	57	58	63	64	67	68	72	73	82	83	91
	0.24	0	11	12	51	53	57	58	63	64	67	68	72	73	82	83	91
	0.25	0	11	12	51	53	57	58	63	64	67	68	72	73	82	83	91
HD3dif	0.0	0	11	12	51	52	58	59	67	68	72	73	82	83	91		
	0.01	0	11	12	51	52	58	59	67	68	72	73	82	83	91		
	0.02	0	11	12	51	52	58	59	67	68	72	73	82	83	91		
	0.03	0	11	12	51	52	58	59	67	68	72	73	82	83	91		
	0.04	0	11	12	51	52	58	59	67	68	72	73	82	83	91		
	0.05	0	11	12	51	52	58	59	67	68	72	73	82	83	91		
	0.06	0	11	12	51	52	58	59	67	68	72	73	82	83	91		
	0.07	0	11	12	51	52	58	59	67	68	72	73	82	83	91		
	0.08	0	11	12	50	52	58	59	67	68	72	73	82	83	91		

0.09	0	11	12	50	52	58	59	67	68	72	73	82	83	91
0.1	0	11	12	50	52	58	59	67	68	72	73	82	83	91
0.11	0	11	12	50	52	58	59	67	68	72	73	82	83	91
0.12	0	11	12	50	52	58	59	67	68	72	73	82	83	91
0.13	0	11	12	50	52	58	59	67	68	72	73	82	83	91
0.14	0	11	12	50	52	58	59	67	68	72	73	82	83	91
0.15	0	11	12	50	52	58	59	67	68	72	73	82	83	91
0.16	0	11	12	50	52	58	59	67	68	72	73	82	83	91
0.17	0	11	12	50	52	58	59	67	68	72	73	82	83	91
0.18	0	11	12	50	52	58	59	67	68	72	73	82	83	91
0.19	0	11	12	49	52	58	59	67	68	72	73	82	83	91
0.2	0	11	12	49	52	58	59	67	68	72	73	82	83	91
0.21	0	11	12	49	52	58	59	67	68	72	73	82	83	91
0.22	0	11	12	49	52	58	59	67	68	72	73	82	83	91
0.23	0	11	12	49	52	58	59	67	68	72	73	82	83	91
0.24	0	11	12	49	52	58	59	67	68	72	73	82	83	91
0.25	0	11	12	49	52	58	59	67	68	72	73	82	83	91
