# Supplementary material for: Activation of the alpha-globin gene expression correlates with dramatic upregulation of nearby non-globin genes and changes in local and large-scale chromatin spatial structure
Source: Epigenetics Chromatin. 2017 Jul 11;10:35. doi: 10.1186/s13072-017-0142-4 (PMC5504709; doi:10.1186/s13072-017-0142-4)
Supplement: Supplementary file 8 — Additional file 8: Supplementary Methods. A detailed description of the C-TALE library preparation. [file 13072_2017_142_MOESM8_ESM.pdf]

SUPPLEMENTARY METHODS

C-TALE library preparation

C-TALE libraries were prepared in two independent biological replicates. At the first step, *in situ* DpnII-Hi-C libraries suitable for sequencing on Illumina HiSeq platform were prepared from each cell type according to previously published protocol [4]. Next, we produced hybridization baits using BACs CH261-93N17, CH261-75C12 and CH261-85E12 (CHORI BACPAC) covering the genomic region chr14:11721574-12436767. BACs purified from overnight shaker-cultures were mixed in an equimolar ratio (totally 5 µg) in 500 µl of sonication buffer (50 mM Tris-HCl pH 8.0, 10 mM EDTA, 0.1% SDS), incubated on ice for 20 min, and the DNA was then sheared to a size of approximately 200-600 bp using a VirSonic 100 (VerTis). The sample was centrifuged at 13400 g for 5 min and concentrated on AMICON Ultra Centrifugal Filter Units (30K). The DNA was purified using Agencourt AMPure XP magnetic beads (Beckman Coulter) and eluted with 42.5 µl of 1.1× T4 DNA ligase buffer (Thermo). 2.5 µl of 10 mM dNTP mixture (Thermo), 2.5 µl of T4 polynucleotide kinase (NEB; 10 U/µl), 2 µl of T4 DNA polymerase (NEB; 3U/µl), 0.5 µl Klenow DNA polymerase (NEB; 5U/µl) were added to the sample, and end repair reaction was carried out for 30 min at 25°C. After that, the DNA was purified using Agencourt AMPure XP magnetic beads. The A-tailing reaction was performed in 50 µl of 1× NEBuffer 2 supplemented with 0.5 mM dATP and 12.5 U of Klenow exo minus enzyme (NEB) for 30 min at 37°C. The DNA was purified using Agencourt AMPure XP beads and eluted with 19 µl of 10 mM Tris-HCl (pH 8.0). Double-stranded Y-shaped BAC-adaptor for the PCR amplification was ligated to the library in 25 µl of 1× T4 DNA ligase buffer supplemented with 1 mM of the adaptor and 5 U of T4 DNA ligase (Thermo) for 2.5 h at 25°C. BAC-adapter sequences are the following (adopted from PMID:21886102): 5`CCATCTCATCCCTGCGTGTCGACTACACTACTCGT 3`, 5`$PO_4$-CGAGTAGTGTTCAGCAAGGCACACAGGGGATAGG3` (complementary regions are underlined). The DNA was purified using Agencourt AMPure XP beads (0.9:1) and eluted with 25 µl of 10 mM Tris-HCl (pH 8.0). Test PCR reactions containing 1 µl of the bait library were performed to determine the optimal number of PCR cycles needed to obtain 10-30 ng/µl of DNA in the PCR mixture. The PCR reactions (volume of each reaction is 25 µl) were performed using KAPA High Fidelity DNA Polymerase (KAPA) and 5`-biotinylated custom PCR primers (12.5 pmol each; forward primer: biotin-CCATCTCATCCCTGCGTGTC; reverse primer: biotin-CCTATCCCCTGTGTGCCTTG). The temperature profile was 5 min at 98°C, followed by 8, 10 and 12 cycles of 20 s at 98°C, 15 s at 65°C, and 20 s at 72°C. 1-2 µg of the final bait library from 3-4 preparative PCR reaction was purified using Agencourt AMPure XP beads and eluted with 10-20 µl of 10 mM Tris-HCl (pH 8.0) to obtain the DNA concentration higher than 50 ng/µl that is handy for the performing of subsequent hybridization.

For hybridization of the Hi-C libraries with the bait library, two starting mixtures (15 µl each) were prepared. The first mixture (Bac-mix) contains 150 ng of the bait library (100 ng per one Hi-C library), 300 ng of sonicated genomic DNA (200-500 bp) and 10 pmol of competitor oligonucleotides for the BAC-adaptor (oligo-A1 5′ CCATCTCATCCCTGCGTGTCGACTACACTACTCGT 3′, oligo-A2 5′ CCTATCCCCTGTGTGCCTTG 3′). The second one (Lib-mix) contains 300 ng of Hi-C libraries from the three cell types mixed in equimolar ratio, 3 µg of salmon sperm DNA and 10 pmol of competitor oligonucleotides for the Illumina TruSeq adaptors (oligo-B1 5’ AATGATACGGCGACCACCGAGATCTACACTCTTTCCCTACACGACGCTCTTCCGATCTC 3`, oligo-B2 is Illumina PE PCR Primer 2.0 5` CAAGCAGAAGACGGCATACGAGATCGGTCTCGGCATTCCTGCTGAACCGCTCTTCCGATCT 3`). Both mixtures were denaturated in a PCR-machine at 95$^{o}$C for 5 min and then incubated at 65$^{o}$C for 15 min. After that, 15 µl of pre-heated (65$^{o}$C) 2× hybridization buffer (10× Denhardt`s solution, 10× SSPE buffer, 0.2% SDS, 10 mM EDTA) were added to the both mixtures, and the tubes were incubated at 65°C for 3 h. Next, BAC-mix was added to the Lib-

mix with pre-heated low-binding tip, and the final hybridization mixture was incubated at 65°C for 40 h (30-50 h is an appropriate time interval).

At the next step, 10 µl of streptavidin-coated magnetic beads Dynabeads C1 (Invitrogen) were washed with 200 µl of WB buffer (10 mM Tris-HCl pH 7.5, 1M NaCl, 1 mM EDTA), resuspended in 150 µl of WB buffer, combined with the hybridization mixture and incubated for 30 min at 25°C with occasional vortexing to pull-down biotinylated baits with trapped ligation junctions. Unbound portion of Hi-C library and other components of the hybridization mixture were removed by sequential washing the beads with 200 µl of LSB buffer (1× SSC, 0.1% SDS) for 15 min at 25°C (single wash), 200 µl of HSB buffer (0.1× SSC, 0.1% SDS) for 10 min at 65°C (three washes) and 200 µl of LS buffer (10 mM Tris-HCl pH 8.0, 10 mM NaCl) for 10 min at 25°C (three washes; hereinafter low-binding tips and tubes were used for all manipulations). After the last washing, the beads were resuspended in 25 µl of 10 mM Tris-HCl pH 8.0. Test PCR reactions containing 5 µl of the bead suspension were performed to determine the optimal number of PCR cycles needed to obtain 10-30 ng/µl of DNA in the PCR mixture. The PCR reactions (volume of each reaction is 25 µl) were performed using KAPA High Fidelity DNA Polymerase (KAPA) and Illumina PE1.0 and PE2.0 primers (12.5 pmol each). The temperature profile was 5 min at 98°C, followed by 17, 19 and 21 cycles of 20 s at 98°C, 15 s at 65°C, and 20 s at 72°C. Two preparative PCR reactions were performed, the PCR mixtures were combined, and the products were purified using Agencourt AMPure XP beads (the volume ration of beads and PCR mixture is 0.8 : 1).

Finally, the obtained library was subjected to the second round of hybridization performed essentially as the first one to achieve at least a 300-fold enrichment of ligation junctions from the locus of interest. 10 cycles of PCR were applied after the second round of hybridization. Final libraries were sequenced on Illumina HiSeq 2000 by 10-12 millions of paired-end reads.
